# Modeling the Metabolism of *Arabidopsis thaliana*: Application of Network Decomposition and Network Reduction in the Context of Petri Nets

**DOI:** 10.3389/fgene.2017.00085

**Published:** 2017-06-30

**Authors:** Ina Koch, Joachim Nöthen, Enrico Schleiff

**Affiliations:** ^1^Department of Molecular Bioinformatics, Institute of Computer Science, Cluster of Excellence “Macromolecular Complexes”, Goethe-University FrankfurtFrankfurt am Main, Germany; ^2^Department of Biosciences, Institute of Molecular Biosciences, Molecular Cell Biology of Plants, Cluster of Excellence “Macromolecular Complexes”, Goethe-University FrankfurtFrankfurt am Main, Germany

**Keywords:** systems biology, Petri net, *Arabidopsis thaliana* metabolism, model verification, network reduction, transition invariant, common transition pairs, invariant transition pairs

## Abstract

**Motivation:** *Arabidopsis thaliana* is a well-established model system for the analysis of the basic physiological and metabolic pathways of plants. Nevertheless, the system is not yet fully understood, although many mechanisms are described, and information for many processes exists. However, the combination and interpretation of the large amount of biological data remain a big challenge, not only because data sets for metabolic paths are still incomplete. Moreover, they are often inconsistent, because they are coming from different experiments of various scales, regarding, for example, accuracy and/or significance. Here, theoretical modeling is powerful to formulate hypotheses for pathways and the dynamics of the metabolism, even if the biological data are incomplete. To develop reliable mathematical models they have to be proven for consistency. This is still a challenging task because many verification techniques fail already for middle-sized models. Consequently, new methods, like decomposition methods or reduction approaches, are developed to circumvent this problem.

**Methods:** We present a new semi-quantitative mathematical model of the metabolism of *Arabidopsis thaliana*. We used the Petri net formalism to express the complex reaction system in a mathematically unique manner. To verify the model for correctness and consistency we applied concepts of network decomposition and network reduction such as transition invariants, common transition pairs, and invariant transition pairs.

**Results:** We formulated the core metabolism of *Arabidopsis thaliana* based on recent knowledge from literature, including the Calvin cycle, glycolysis and citric acid cycle, glyoxylate cycle, urea cycle, sucrose synthesis, and the starch metabolism. By applying network decomposition and reduction techniques at steady-state conditions, we suggest a straightforward mathematical modeling process. We demonstrate that potential steady-state pathways exist, which provide the fixed carbon to nearly all parts of the network, especially to the citric acid cycle. There is a close cooperation of important metabolic pathways, e.g., the *de novo* synthesis of uridine-5-monophosphate, the γ-aminobutyric acid shunt, and the urea cycle. The presented approach extends the established methods for a feasible interpretation of biological network models, in particular of large and complex models.

## 1. Introduction

*Arabidopsis thaliana* (*A. thaliana*) is a popular model organism in plant biology (Van Norman and Benfey, 2009). *A. thaliana* was the first plant with sequenced genome (Arabidopsis-Genome-Initiative, 2000), and a large mutant collection (Sessions et al., 2002; Alonso et al., 2003) provides the optimal base for genetic and physiological analysis of this model system. It further is characterized by a short generation time, small plant size, diploid genetics, and a large number of offspring, which is of high advantage for breeding for research (Meinke et al., 1998; Koornneef and Meinke, 2010 and references therein). Most of the current information on plant metabolism is based on this model system (Lunn, 2007). Beside the academic interest in understanding the metabolism of plants, there is a broad interest to improve nutritional quality of crops and agricultural productivity to generate phytopharmaceutical substances and to increase the production of nutraceutical biomolecules and pharmaceutical proteins of commercial interest (Hur et al., 2013). The development of network models based on these investigations (Dersch and Beckers, 2016) represents a useful approach for the analysis and simulation of the metabolism. Nowadays, the Path2Models database contains 125 models for *A. thaliana* (Büchel et al., 2013). To develop a mathematical model, the single reactions are typically extracted from databases such as AraCyc (Mueller et al., 2003; Zhang et al., 2005), and/or KEGG (Kanehisa et al., 2008).

Various models of the metabolism of *A. thaliana* exist. Several approaches concern gene regulatory networks (Lucas and Brady, 2013). For example, a method based on Bayesian networks was formulated to investigate root cell differentiation (Bruex et al., 2012), and a network, focusing on stress response in leaves, was developed (Hickman et al., 2013). The power of these networks has been documented, for example, by identification of new factors involved. Using a recently developed statistical linear regression technique, novel genes were detected which are involved in the mucilage biosynthesis (Vasilevski et al., 2012).

In this paper, we focus on recent research results of metabolic modeling approaches of *A. thaliana*. The first steady-state *Metabolic Flux Analysis (MFA)* maps for *A. thaliana* were introduced in 2008 (Williams et al., 2008), using a heterotrophic cell suspension grown under two different oxygen concentrations as experimental data source. The results suggest a possible alteration of metabolite abundance without changes in the balance between respiratory and biosynthetic flux or a major rearrangement of the network. Based on this study, further investigations have been followed, in particular on the flux in the pentose phosphate pathway (Masakapalli et al., 2010). Three new models were derived, which differ in the compartmental organization of the pentose phosphate pathway. The measured data fit to each of the three models in an acceptable manner, which necessitate further investigations in addition to the MFA. This underlines the problems of metabolic flux analysis (Masakapalli et al., 2010). Several genome-scale flux models have been developed for *A. thaliana*, for example, by Poolman et al. (2009), de Oliveira Dal'Molin et al. (2010), Radrich et al. (2010), and Mintz-Oron et al. (2012).

The *Poolman model* is based on the AraCyc database (Mueller et al., 2003) and was automatically extracted. It consists of 1,253 metabolites and 1,406 reactions, involving the production of biomass components, such as nucleotides, amino acids, lipid, starch, and cellulose in the proportion experimentally observed in a heterotrophic suspension culture. After the removal of reactions that are not necessary to maintain a steady state, 855 reactions remain. Additionally, the authors provide a steady-state model of 232 reactions that exhibit only nonzero flux values.

Similarly, de Oliveira Dal'Molin et al. (2010) automatically extract a core reaction system called AraGEM from the KEGG database to generate a model. The model is compartmentalized into cytosol, mitochondrion, plastid, perixome, and vacuole. Additional information is manually integrated from databases, such as AraPerox (Reumann et al., 2004) and SUBA (Arabidopsis Subcellular Database, Heazlewood et al., 2007). The model contains 1,567 reactions and 1,748 metabolites. A two-dimensional annotation provides links from the reactions to the genes. The authors modify 36 reactions by manual curation to give a consistent stoichiometry. To achieve a desired functionality, they introduce 148 biomass drains and inter-organelle transporters.

The *Radrich model* represents a high-quality core consensus model obtained by a systematic comparison of compounds and reactions between the databases KEGG and AraCyc. Various levels of consensus lead to three submodels of different quality, the core model of 753 reactions and 914 metabolites with highest reliability, the intermediate model of 1,388 reactions and 1,248 metabolites, and the complete model of 2,315 reactions and 2,328 metabolites. The core model is the intersection between the two databases, containing every metabolite and reaction, which is present in both databases. In the intermediate model, every reaction is present, for which either all educts or all products are part of the core model. The complete model is the union of both databases. The *Mintz-Oron model* was semi-automatically derived from KEGG and AraCyc, including compartmental information from the Arabidopsis Subcellular Database, SUBA (Heazlewood et al., 2007), and tissue-specific localization data from the literature. It consists of 1,363 reactions and 1,078 metabolites. The authors predicted a total of 942 out of 1,363 inspected reactions to take place in every tissue. These reactions include reactions of the primary metabolite pathways, such as the glycolysis, the pentose phosphate pathway, and the fatty acid, nucleotide, and amino acid metabolism. The model is validated by comparison of experimental data with predicted flux values. All presented models use the databases AraCyc and/or KEGG as starting points for automatic network generation.

We chose Petri nets (PNs) as mathematical formalism for network construction, analysis, and simulation. PNs are mainly applied in computer science, for example, to model distributed systems. Many sound, rigorous analysis and simulation methods of PNs have been evolved over decades for various applications (Billington and Reisig, 1996). Some of these concepts and algorithms have been successfully applied to biochemical systems, including metabolic networks (Koch et al., 2005), signal transduction networks (Sackmann et al., 2006), gene regulatory networks (Matsuno et al., 2000), protein complex assembly (Bortfeldt et al., 2011), and combinations of them (Grunwald et al., 2008; Koch et al., 2011).

To motivate the work, we aimed to develop a consistent metabolic model that reflects the steady-state condition and the basic dynamic behavior. Thus, the model becomes suitable for rigorous mathematical analyses and can serve as basis for quantitative analyses. Our work was motivated by a hand-built PN model for the metabolism of barley *Hordeum vulgare*, integrating biochemical, physiological, proteomic, and genomic data derived from the literature and databases (Grafahrend-Belau et al., 2009). We followed the paper's strategy and built a model for *A. thaliana* by successively adding metabolites and reactions from the literature (Nöthen, 2009). We systematically expanded the PN to develop a new model based on the current experimental knowledge on the metabolism of *A. thaliana* and to investigate the structural and dynamic properties of the model. To check a metabolic model for consistency and correctness, we considered special network properties (Heiner and Koch, 2004). For biochemical systems, a correct biological interpretation of submodules at steady state was mainly applied. The computation of such submodules was based on minimal semi-positive transition invariants (TIs), also known as elementary modes (Schuster and Hilgetag, 1994). For the definition, see Section 2.1. Minimal semi-positive integer solutions were of interest (Schrijver, 1998; Koch and Ackermann, 2013) leading to a complexity that does not allow the computation of all solutions, even if the the power of supercomputers is used. Today, several alternative methods for the exploration of the inherent flux states of large systems exist (Koch and Ackermann, 2013).

The paper is organized as follows. In the Section 2, we introduce Petri nets and describe the network verification and the network reduction techniques we used, including the common transition pairs and invariant transition pairs. In Section 3, we give the complete PN and the reduced PN model of the central metabolism in *A. thaliana*. We explain an example for a network reduction and for a TI, and discuss the *Maximal Common Transition sets*, covering the analyses of functional modules. The supplementary file Table 1.pdf contains the metabolites, the reactions, and the output reactions, including the literature references. The supplementary file Table 2.pdf contains the tables, indicating each reduction step. The supplementary file Supplementary Material Data Sheet 1 includes all Petri net models.

## 2. Materials and methods

In the following, we briefly describe the methods for network reduction and network decomposition given in the Petri net formalism.

### 2.1. Petri nets

Petri nets (PNs) are based on a concept of communication of automation originally introduced by Carl Adam Petri (Petri, 1962) in his dissertation in 1962 for mathematical modeling of causal systems with concurrent processes. PN provide a flexible, well-defined, mathematical formalism for various modeling types, ranging from qualitative to quantitative modeling. Here, we introduce the basic definitions and notations that are necessary to understand the paper. For a more detailed introduction, see Murata (1989), Baumgarten (1996), and Koch et al. (2011).

The first biological application of PNs was published in 1993 by Reddy et al. (1993). PNs has been used to model various biochemical systems, such as metabolic networks (Koch et al., 2005), signal transduction networks (Sackmann et al., 2006), gene regulatory networks (Matsuno et al., 2000), protein complex assembly (Bortfeldt et al., 2011), and combinations of them (Grunwald et al., 2008). The PN formalism has been also used for stochastic modeling, applying the Gillespie's algorithm (Gillespie, 1977), and for kinetic modeling, applying mass action kinetics and/or Michaelis-Menten kinetics. For a review, see Koch et al. (2011).

Petri nets are directed, bipartite, labeled graphs. They consist of two disjunctive sets of vertices, *P* and *T*, respectively. The elements in *P* are the *places* graphically represented as circles, and the elements in *T* are the *transitions* graphically represented as rectangles. Places stand for the passive system's elements, for example, chemical compounds, metabolites, proteins, and protein complexes. Instances of them are represented by *tokens*, which define discrete entities. Since the movement of tokens realizes the dynamics of a PN, we introduce the concept of tokens in Section 2.1.1. Transitions are the active system's elements, for example, chemical reactions, degradation processes, and complex assembly processes. Places and transitions are connected by directed, labeled edges. Edges between vertices of the same type are not allowed. Usually, the edges are labeled or weighted, respectively, by integer numbers. In the following, we call a directed, bipartite graph *net* or *net graph*, see Definition 2.1. To define a Petri net, we extend that definition, compare Definition 2.2.

**Definition 2.1. Net or net graph**.

A net or net graph is a triple *N* = (*P, T, F*) with
*P* ∩ *T* = ∅ and
*F* ⊆ (*P* × *T*) ∪ (*T* × *P*)).

We call the elements of *P* and *T* places and transitions, respectively. They are the vertices or nodes of the net. We call the elements of the flow relation, *F*, edges or arcs.

Regarding a net vertex, we define two sets of neighbor vertices, the set of all pre-vertices •*x*: = {*y* | (*y, x*) ∈ *F*} and the set of all post-vertices *x*•: = {*y* | (*x, y*) ∈ *F*}. Accordingly, we write for a set of *pre-places*, •*t*, for a set of *post-places*, *t*•, for a set of *pre-transitions*, •*p*, and for a set of *post-transitions*, *p*•.

We call edges that are going in two opposite directions as *read arcs* or *test arcs*. Using read arcs, we can model, for example, catalytic reactions, where the catalyst is necessary to activate the transition (reaction), but will not be consumed, when the transition takes place. A PN without read arcs is a *pure* PN.

**Definition 2.2. Petri net**. A Petri net or Place/Transition net or P/T net is a six-tuple *Y* = (*P, T, F, K, W, M*_0_), if

- (*P, T, F*) is a net,
- *K* : *P* → ℕ ∪ {∞} (capacities of the places, possibly infinite),
- *W* : *F* → ℕ (edge weights or label of the edges), and
- *M*_0_:*P* → ℕ_0_ (initial marking) with ∀*p* ∈ *P* : *M*_0_(*p*) ≤ *K*(*p*).

#### 2.1.1. The dynamics of P/T nets

The dynamics of a net is performed by movable objects called *tokens* which are located on the places and will be removed according to the *firing rule*. If *N* is a P/T net, a mapping of the *M* : *P* → ℕ_0_ with ∀*p* ∈ *P* : *M*(*p*) ≤ *K*(*p*) is called a marking of *N*. We graphically represent the number of tokens of a place, *M*(*p*), under a marking, *M*, by *M*(*p*) dots (tokens) on the place, *p*. We write the capacities ≠∞ and edge weights ≠1 at the places and edges, respectively. The token distribution over all places define a certain state of the net. M(N) is the set of all markings of *N*. In the following, let *M* be a fixed marking of *N*. The number of the tokens can be restricted by the capacity of the place. In most biological applications, the capacity is set to infinite. Additionally, we introduce *logical* vertices to get an improved layout. A logical vertex has copies of the same name in the graphical representation of the model. Using logical vertices, we can draw PNs in a clearly-arranged way. For example, in metabolic networks, ATP participates in many reactions. This leads to many crossing edges if we model only one place for ATP. To avoid these crossing edges, we copy the place for ATP and mark it as logic place.

In this paper, we consider the classical *Place/Transition PN (P/T net)*. The firing rules do not include time relations. A transition *fires*, or for biochemical networks, a reaction takes place, if the transition is *activated* or *has concession*, i.e., if the pre-places carry at least as many tokens as indicated by the weights of the corresponding edges and if the capacity of the post-places is large enough as indicated by he corresponding edge weights. At the moment of firing, the tokens of the pre-places will be consumed, and the tokens on the post-places will be produced, both according to the corresponding edge weights. A new system's state is achieved. The numbers of tokens and the edge weights implement quantitative properties although the system is still discrete. If always at least one transition of the PN is activated, the PN contains no deadlock and is called to be *deadlock-free*.

**Definition 2.3. Activated transition**. A transition, *t* ∈ *T*, is activated or has concession under *M*, written as $M\overset{t}{\to }$, if

- ∀*p* ∈ •*t* : *M*(*p*) ≥ *W*(*p, t*),
- ∀*p* ∈ *t*•:*M*(*p*) ≤ *K*(*p*) − *W*(*t, p*).

We say that *t* fires from *M* to *M*′ and write $M\overset{t}{\to }M^{\prime }$, if *t* is activated under *M*, and *M*′ arises from *M* by removal of tokens from the pre-places and production of tokens on the post-places according to the corresponding edge weights:

(1) $$\begin{array}{l} M^{\prime }\left(p\right)=\left\{ \begin{array}{l} M\left(p\right)-W\left(p,t\right),\;if\;p\in •t\;\backslash \;t•,\;\\ M\left(p\right)+W\left(t,p\right),\;if\;p\in t•\;\backslash \;•t,\;\\ M\left(p\right)-W\left(p,t\right)+W\left(t,p\right),if\;p\in t•\cap •t,\;\\ M\left(p\right)\;otherwise. \end{array}\right. \end{array}$$

**Figure 1:**
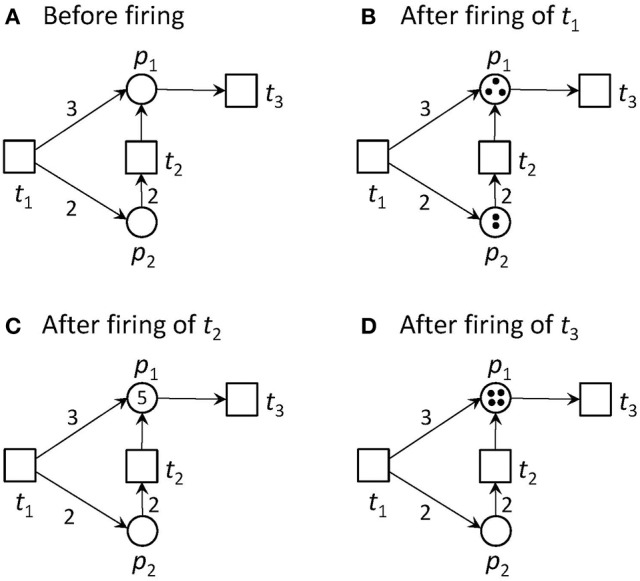
A Petri net and its firing possibilities. **(A)** The PN consists of two places and three transitions. Place *p*_2_ is pre-place of *t*_2_, and *p*_1_ is post-place of *t*_2_. *t*_1_ and *t*_2_ are pre-transitions of *p*_1_, and *t*_3_ is post-transition of *p*_1_. Transition *t*_1_ is an input transition, meaning that it has no pre-places and is, thus, always activated. Transition *t*_3_ is an output transition, meaning that it has no post-places. In the first step, only transition *t*_1_ is activated. Transition *t*_2_ would need two tokens on place *p*_2_ to become activated, and *t*_3_ would need one token to get concession. The capacities of all places are infinite. **(B)** The new state defined by the token distribution after firing of *t*_1_. Place *p*_1_ gets three tokens and *p*_2_ two tokens. Additionally to *t*_1_, *t*_2_ and *t*_3_ are activated, because their pre-conditions defined by the edge weights are fulfilled, and the post-condition is valid too. **(C)** The new state after firing of *t*_2_. For the reachability analysis, also the case of firing *t*_3_ first, will be considered. In the simulation, one of the activated transitions is randomly chosen to fire next. Now, *t*_1_ and *t*_3_ are activated. **(D)** The new state after firing of *t*_3_. One token was removed from the PN, because *t*_3_ has no post-place.

Transitions without pre-places are always activated. We call them *input transitions*. Accordingly, we call transitions without post-places *output transitions*. Input and output transitions were used to model the interface to the system's environment. Figure 1 illustrates an example of a pure PN and its firing behavior.

#### 2.1.2. Linear invariants

Using linear invariants, we can define specific dynamic properties of the network. These properties are valid in every system state. They can be used for model verification, but also for model decomposition and network reduction. For PNs, we define the *place invariants (PI)* for the passive part and the *transition invariants (TI)* for the active part. The definitions of both invariant types are based on the incidence matrix, we also know as stoichiometry matrix for metabolic networks. This *m* × *n* matrix, *C*, for *m* places and *n* transitions indicates for each place, *p*_*i*_ ∈ *P*, the change in the number of tokens, △*m*, when a transition, *t*_*j*_ ∈ *T*, fires.

**Definition 2.4. Incidence matrix**. Let *N* be a P/T net. The corresponding incidence matrix *C* is defined by: ∀ 1 ≤ *i* ≤ *m*, 1 ≤ *j* ≤ *n*

(2) $$\begin{array}{ll} C_{i,j}= & \{\begin{array}{l} W\left(t_{j},p_{i}\right),\;if\;\left(t_{j},p_{i}\right)\in F\;\backslash \;F^{-1},\;\\ -W\left(p_{i},t_{j}\right),if\;\left(p_{i},t_{j}\right)\in F\;\backslash \;F^{-1},\;\\ W\left(t_{j},p_{i}\right),\;if\;\left(t_{j},p_{i}\right)\in F\cap F^{-1},\;\\ 0\;otherwise. \end{array} \end{array}$$

Note that for a PN without read arcs, we can remove \*F*^−1^ and the third condition. Contrary, for all cases, the third case, *C*_*i, j*_ = *W*(*t*_*j*_, *p*_*i*_), *if*(*t*_*j*_, *p*_*i*_) ∈ *F*, would be sufficient, if we consider *W* as complete mapping to (*P* × *T*) ∪ (*T* × *P*) and set the weights of non-existing edges to 0.

We developed the software tool *MonaLisa* especially designed for PN applications to biological systems (Einloft et al., 2013). Additionally to an intuitive editor, the tool provides many useful analysis functions based on PNs as well as on graph theory. It allows for classical discrete modeling as well as for stochastic modeling (Balazki et al., 2015).

**Definition 2.5. Place invariant (PI)**. Let *N* be a P/T net and *C* the corresponding incidence matrix. A place invariant of *N* is an *m*-tuple *x* ∈ ℤ^*m*^ with *C*^*T*^*x* = 0.

**Definition 2.6. Transition invariant (TI)**. Let *N* be a P/T net and *C* the corresponding incidence matrix. A transition invariant of *N* is an *n*-tuple *y* ∈ ℤ^*n*^ with *Cy* = 0.

We are interested in the minimal, semi-positive, integer solutions; *integer*, because we are working at discrete level, *semi-positive* because there is no interpretation of negative solutions, and *minimal* because such equation systems can have an infinite number of solutions. In the following, we consider minimal, semi-positive, integer PIs and TIs, writing shortly PIs and TIs, respectively. For discussion of the computational problem, see Schrijver (1998) and Koch and Ackermann (2013).

The support of a vector, *x*, is represented by the non-zero entries of the vector written as *supp*(*x*). An invariant *x* is called *minimal*, if its support does not contain the support of any other invariant *z*, i.e.,

(3) $$\begin{array}{l} ∄\text{ invariant }z:supp\left(z\right)\subset supp\left(x\right), \end{array}$$

and the greatest common divisor of all non-zero entries of *x* is one. Whereas PIs reflect a token or substance conservation, the TIs describe basic functional modules of the system's dynamics at steady state (Lautenbach, 1973; Schuster and Hilgetag, 1994; Schuster et al., 2002). These functional modules have to be checked for their biological correctness. The firing of the transitions of a TI in the given frequency reproduces the initial state. Thus, a TI is also called a *Parikh* vector. If every place or every transition, respectively, is member of at least one PI or TI, respectively, the PN is *covered by PIs (CPI)* or *covered by TIs (CTI)*, respectively. The CTI property indicates the network's completeness or consistency. A transition which is not member of at least one TI does not contribute to the systems behavior. Thus, it could be removed without influencing the overall systems behavior. A TI or PI, respectively, defines a connected subnet, consisting of its support, the support's pre- and post-places or pre- and post-transitions, respectively, and all edges in between.

TIs can be classified according to the type of the participating transitions. The classes of TIs are motivated by the involved input and output transitions. We consider the following types of TIs:

- trivial: a reversible reaction, which is split into a forward and a backward transition.
- INOUT: contains at least one input transition and one output transition.
- IN: contains an input transition, but no output transition.
- OUT: contains an output transition, but no input transition.
- CYC: contains neither an input nor an output transitions forming a cycle in the model.

The explanations for some of the different types of invariants are intuitive. In a metabolic PN, containing reversible reactions, it is not possible to avoid trivial TIs. TIs of the type INOUT represent pathways through the network, a succession of consecutive biochemical reactions, transforming given educts (metabolites produced by input transitions) to the corresponding products (metabolites consumed by output transitions). TIs of the types IN or OUT contain only input or output transitions, respectively. These TIs emerge from internal production or consumption, respectively, of secondary metabolites. TIs of type CYC contain neither input nor output transitions at all. Their firings form cycles in the PN.

**Figure 2:**
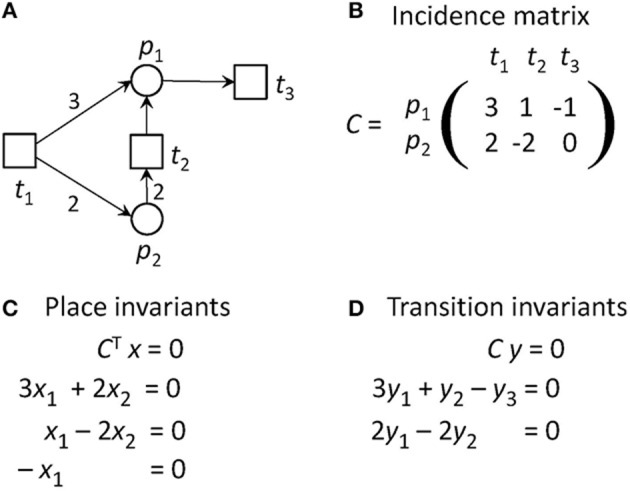
The definitions of invariants of the PN in Figure 1A. **(A)** The PN in Figure 1A. **(B)** The incidence matrix, *C*, of the PN. The matrix indicates how many tokens are removed from or consumed on the places when a transition fires. For example, if *t*_1_ fires, three tokens will be produces on *p*_1_ and two tokens on *p*_2_. **(C)** The equation system to compute the place invariants. **(D)** The equation system to compute the transition invariants. The PN has no place invariants, but is covered by transition invariants. It has one TI = {*t*_1_, *t*_2_, 4 *t*_3_}, meaning that *t*_1_ and *t*_2_ have to fire each once and *t*_3_ four times, before the original state will be reached again.

Figure 2 gives the incidence matrix and the equation systems for PI and TI computation of the PN example of Figure 1.

#### 2.1.3. Maximal common transition sets (MCT-sets)

To support the biological interpretation of TIs, we group the transitions into *Maximal Common Transition sets (MCT-sets, MCTS)* by their occurrence in the minimal TIs: ∀*i, j* ∈ {1, …, *m*} the transitions, *t*_*i*_ and *t*_*j*_, are grouped into the same MCT-set, if and only if they participate in exactly the same minimal TIs, i.e., all TIs *x* hold:

(4) $$\begin{array}{l} \chi_{\left\{0\right\}}\left(x_{i}\right)=\chi_{\left\{0\right\}}\left(x_{j}\right), \end{array}$$

whereas χ_{0}_ denotes the characteristic function, binary indicating whether an argument is equal to zero. This grouping leads to maximal sets of transitions, whereat each set of transitions ϑ holds:

(5) $$\begin{array}{l} \forall x\in X:\;\vartheta \subseteq supp\left(x\right)\;\dot{\vee }\;\vartheta \cap supp\left(x\right)=\emptyset , \end{array}$$

whereas *X* denotes the set of all minimal TIs, *x*.

This grouping represents an equivalence relation in *T*, the set of transitions, which leads to a partition of *T*. The equivalence classes ϑ correspond to the MCT-sets. MCT-sets define also subnets as TIs, but they have not to be connected. The subnets defined by MCT-sets are disjunctive. They represent a further decomposition method of large biochemical networks into rather small subnets at steady state, which often correspond to functional units. Each of the MCT-sets may represent a building block with a special biological meaning.

### 2.2. Network verification

Network verification is a crucial part in the process of model construction. For biological PNs, beside structural and behavioral properties such as the connectivity (Heiner and Koch, 2004; Koch et al., 2011), another important property is the biological interpretability of the TIs (Heiner and Koch, 2004; Koch et al., 2005; Grunwald et al., 2008) and the MCT-sets (Sackmann et al., 2006; Grafahrend-Belau et al., 2008).

The CTI property results from the TIs. This property can be seen as one indicator for the consistency and completeness of a network. It is of particular importance that a biological network is CTI, because this property ensures that each reaction may contribute to the basic system behavior (Koch and Heiner, 2008) while the steady state of the system is preserved.

We assume that metabolic networks reach a steady state. This steady-state assumption is reflected in the computation of the TIs. A TI represents a set of reactions whose enzymes ensure the steady-state condition. Only one missing reaction would lead to a disturbance of the steady state of the system. Moreover, each TI should represent a functional module in the overall network dynamics. The verification or interpretation for a biological meaning of each functional module, i.e., of each TI, represents a method to verify large networks with regard to their correctness. In this context, the computability of all TIs is a big problem, for even middle-sized networks, i.e., consisting of some hundreds of vertices and thousands of edges (Ackermann and Koch, 2011). To handle this problem, we applied network reduction techniques.

#### 2.2.1. Network reduction

Network reduction methods should reduce the complexity of the system, conserving main properties and the main behavior of the network. Reduction techniques are essential for network verification and analysis of big, complex systems. In computer science, the question for the correctness of algorithms, their running times, and other theoretical aspects of the reduction process are of great interest (Arnborg et al., 1993). Reduction has been employed in the analysis of complex networks in the Petri net community during the last decades. Various methods for the structural reduction of series of transitions and places have been developed (Lee-kwang et al., 1987). In this reduction process, surrounding vertices were summarized under conservation of special PN properties. Other techniques rely on the sharing properties of two or more vertices. Parallel transitions or places are connected to the same sets of pre-places and post-places or pre-transitions and post-transitions, respectively. Then, these parallel structures can be merged (Murata, 1989). Other methods try to integrate deadlock-avoiding policies, for example, in flexible manufacturing systems (Uzam, 2004). Beside these structural reduction methods, there exist also dynamical techniques for PNs (Berthelot, 1987).

In systems biology, reduction techniques are used at the topological level as well as at steady-state level. All these techniques help to verify and analyze biochemical networks. Investigations on metabolic networks (Reddy et al., 1993) apply reduction techniques of parallel structures. One approach establishes a matrix-based method to study the hierarchical organization of metabolic networks (Ravasz et al., 2002). To reduce the size of the matrix, non-branching pathways in the metabolic network of *Escherichia coli* are replaced by single reactions, resulting in a decrease of complexity.

**Figure 3:**
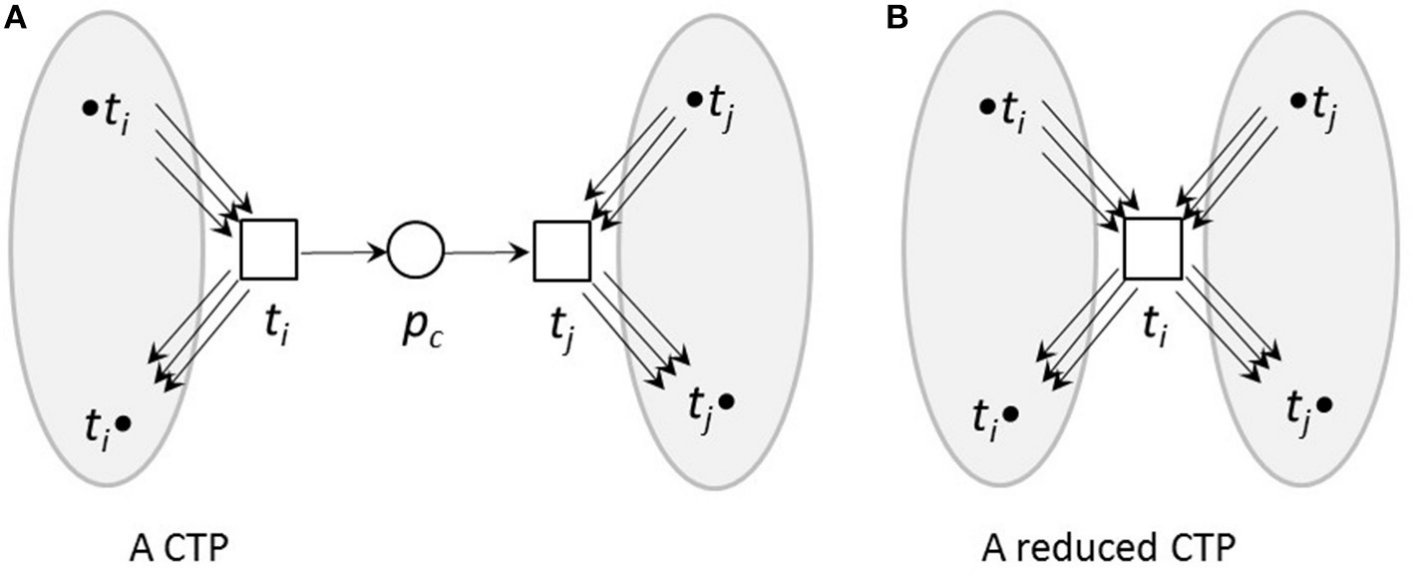
A CTP **(A)** and its reduced form **(B)**. **(A)** A common transition pair, CTP, (*t*_*i*_, *t*_*j*_). The connecting place *p*_*c*_ has one pre-transition *t*_*i*_ and one post-transition *t*_*j*_. **(B)** The net after a CTP reduction. The transition *t*_*j*_ as well as the connecting place *p*_*c*_ were deleted. The transition *t*_*i*_ absorbed the properties of the deleted transition *t*_*j*_, i.e., its pre-places, •*t*_*i*_, and post-places, *t*_*i*_•, as well as the corresponding edges.

Here, we used topological reduction techniques as well as steady-state reduction techniques. We considered topological reduction techniques that preserve the CTI-property (Ackermann et al., 2012). To reduce chains of transitions, we defined *Common Transition Pairs (CTPs)* as two transitions, *t*_*i*_ and *t*_*j*_, connected by a place called a *connecting place*, *p*_*c*_, see Figure 3. We deleted the transition *t*_*j*_ as well as the connecting place, *p*_*c*_. The transition, *t*_*i*_, absorbed the properties of the deleted transition, *t*_*j*_, i.e., all its pre-places, •*t*_*i*_, and post-places, *t*_*i*_•, as well as the corresponding connecting edges.

**Figure 4:**
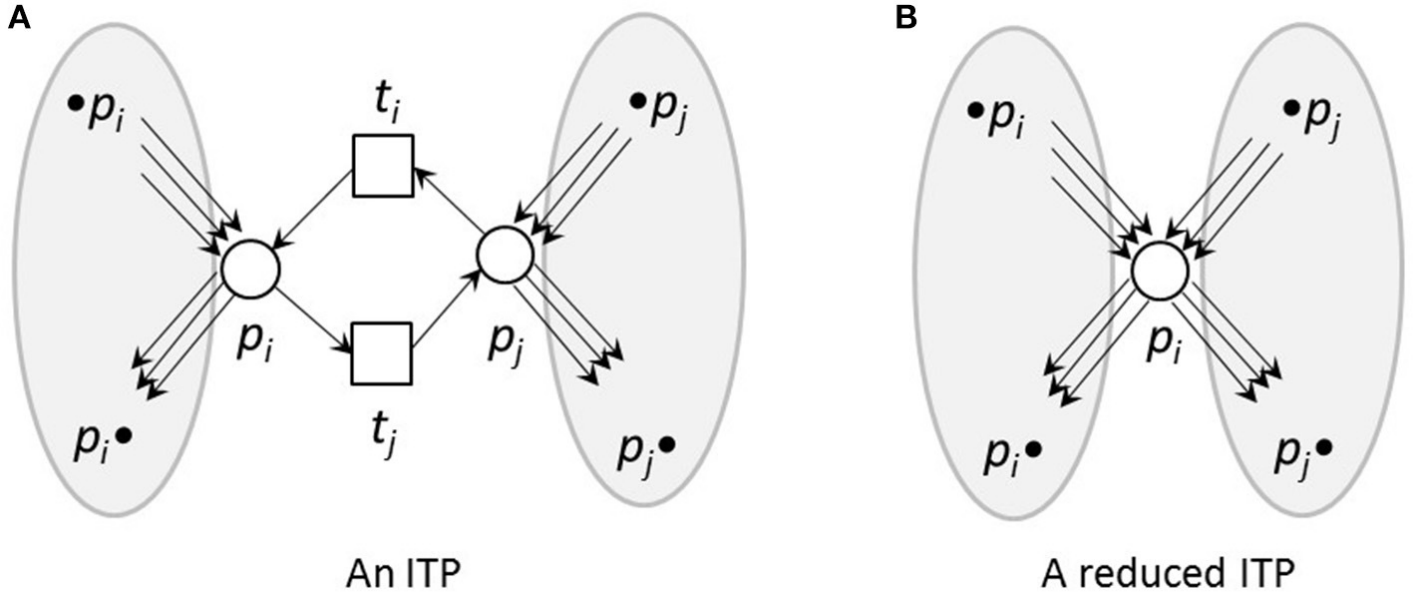
An ITP **(A)** and its reduced form **(B)**. **(A)** An invariant transition pair, ITP (*t*_*i*_, *t*_*j*_). Both transitions have exactly one pre-place and one post-place. The post-place of transition *t*_*i*_ is the pre-place of transition *t*_*j*_ and vice versa. **(B)** Net reduction of an ITP. The ITP *t*_*i*_, *t*_*j*_ as well as the place *p*_*j*_ were deleted. The remaining place *p*_*i*_ absorbed the properties of the deleted place *p*_*j*_, i.e., its pre-transitions, •*p*_*i*_ and •*p*_*j*_, and post-transitions,*p*_*i*_• and *p*_*j*_•, as well as the corresponding edges and markings.

**Figure 5:**
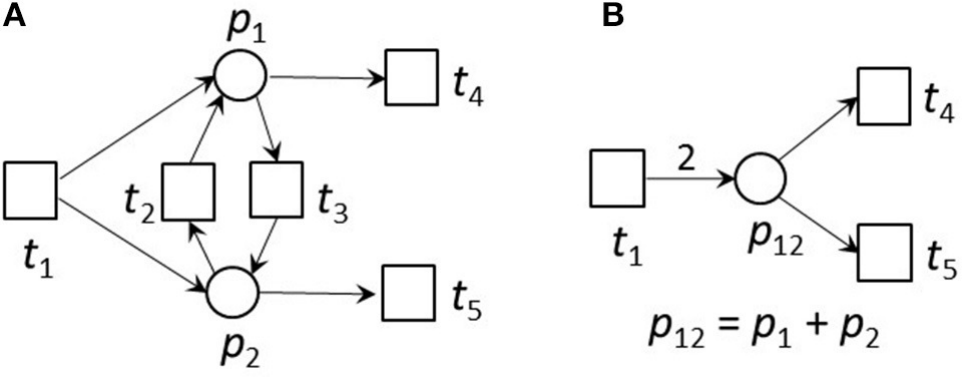
An example for an ITP **(A)** and its reduced form **(B)**. **(A)** An example of a small network that is CTI and contains an ITP (*t*_*i*_, *t*_*j*_). **(B)** The reduced network of **(A)**. Place *p*_12_ absorbed the edges of places *p*_1_ and *p*_2_ of the original net. The transitions *t*_2_ and *t*_3_ were neglected. The weight of edge *t*_1_, *p*_12_ was the sum of the weights of the edges *t*_1_, *p*_1_ and *t*_1_, *p*_2_ of the unreduced net in **(A)**. The reduced net has the two TIs, (*t*_1_ + 2*t*_4_) and (*t*_1_ + 2*t*_5_). Extensions of these invariants gave the TIs,(*t*_1_ + *t*_3_ + 2*t*_4_) and (*t*_1_ + *t*_2_ + 2*t*_5_) of the original net. The TI, (*t*_1_ + *t*_4_ + *t*_5_), is not minimal for the reduced net and will not be generated.

To reduce reversible reactions, we defined *Invariant Transition Pairs (ITPs)* as two transitions, *t*_*i*_ and *t*_*j*_, representing the forward and backward reaction, respectively, of a reversible reaction and connecting two places, *p*_*i*_ and *p*_*j*_, see Figures 4, 5 for an example. We deleted the ITP (*t*_*i*_, *t*_*j*_), as well as the place *p*_*j*_. The place *p*_*i*_ absorbed the properties of the deleted place *p*_*j*_, i.e., all its pre-transitions, •*p*_*i*_, and post-transitions, *p*_*j*_•, as well as the corresponding connecting edges and the markings.

To indicate a reduction, we changed in each reduction the names of the reactions and the metabolites. If two reactions were merged in a CTP reduction, we provided a new name for the merged transition, e.g., “*ctp*(*t*_*i*_+*t*_*j*_)”. If we identified, for example, the two reactions *E*151 and *E*152 connected by metabolite 87 as a potentially reducible CTP, we connected all edges from reaction *E*152 to reaction *E*151, removed metabolite 87 and reaction *E*152, and renamed reaction *E*151 to *ctp*(*E*151 + *E*152).

If the reduction was based on an ITP, we renamed the merged metabolites. According to the CTP reduction, we connected the names of the two metabolites in the same way preceeded by an ITP. If we reduce, for example, the reactions *E*24_*f* and *E*24_*b*, which connect the metabolites 76 and 50, by an ITP reduction, we would connect all edges from metabolite 50 to metabolite 76, remove the reactions *E*24_*f* and *E*24_*b* and the metabolite 50, and rename metabolite 76 to *itp*(76 + 50).

Additionally, we reduced parallel reactions, i.e., if two transitions had the same input place and the same output place, and the same weights on the corresponding edges, we combined both transitions into one without changing the net behavior (Murata, 1989). We adopted this rule for the analysis of biochemical networks (Reddy et al., 1993). We used this technique to further reduce the complexity of the model by reducing parallel pairs of transitions, but we applied it to those with only one pre-place and one post-place and edges of weight 1. The reduction procedure was recursive. It was possible that, e.g., the place *itp*(76 + 50) could be identified as an ITP with another place in the next step. Each step of the reduction could be followed by the nomenclature and produced a timescale of reductions, compare with the Tables 1–9 in the supplementary file Table 2.pdf.

A place or transition that had no connections left was removed from the model. It was possible to remove the just reduced CTP, i.e., the transition *t*_*i*_ ∈ *T*, if the transition pair, (*t*_*i*_, *t*_*j*_], was additionally a reversible reaction for all other places connected to it. When this situation appeared, each ingoing edge for each connected place would cancel an outgoing edge of the same place, and, therefore, cancel all edges from and to *t*_*i*_ ∈ *T*. After an ITP reduction, it was possible that another ITP was removed, if it connects the same *p*_*i*_ and *p*_*j*_. For every other potential ITP, the reduction of the first ITP resulted into two transitions connected to the reduced place *p*_*i*_ ∈ *P* with an ingoing and outgoing edge. We then removed these transitions, because they were no longer connected to the model.

## 3. Results and discussion

### 3.1. The petri net model

**Figure 6:**
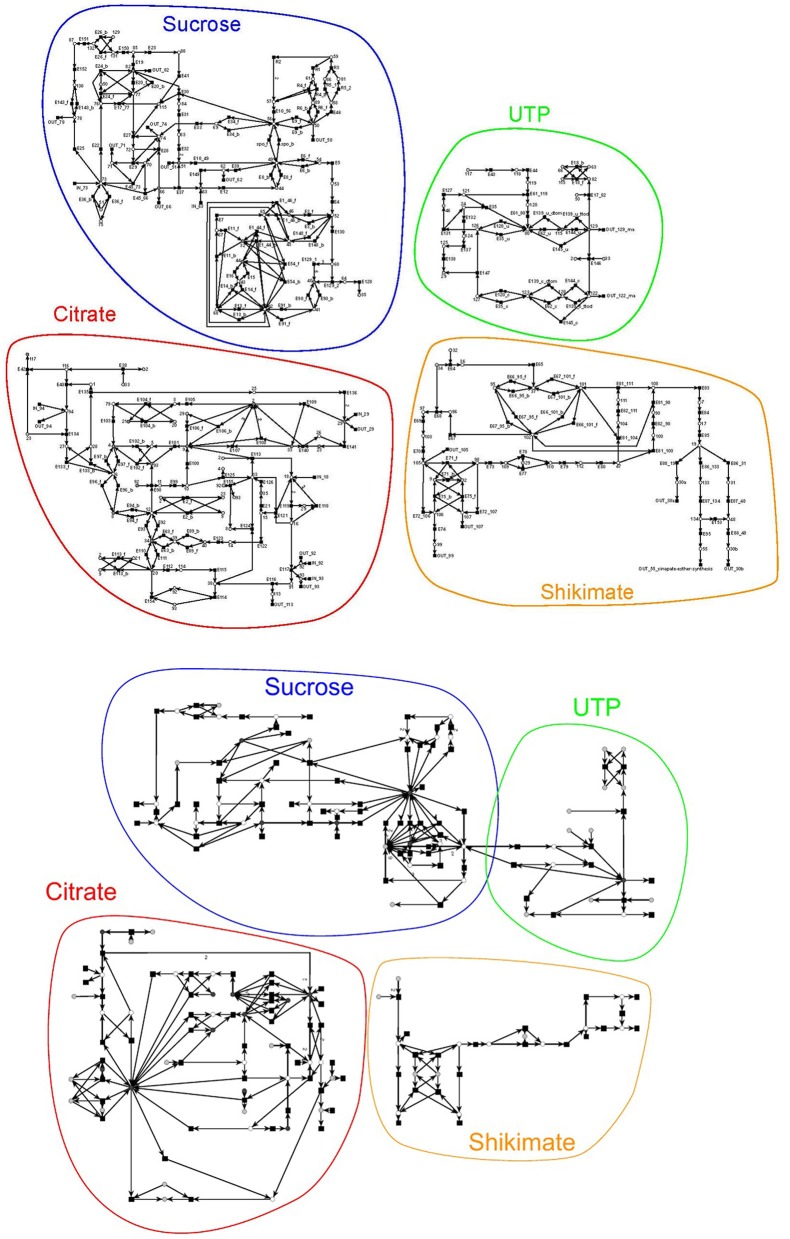
Schemas of the original and reduced PN model adapted from Nöthen (2014). It is a coarse-grained representation of the metabolism of *A. thaliana*. The original model (on the top) consists of 134 metabolites and 242 reactions connected via 569 edges and the reduced model (on the bottom) of 60 metabolites, 131 reactions, and 329 edges. The models were manually subdivided into four subnets, the sucrose, the UTP, the citrate, and the shikimate net. The subnets are highlighted in the same color and name.

We developed the PN model based on the literature, i.e., each reaction (transition) is experimentally proven. The complete PN model consists of 134 metabolites and 243 reactions, which are connected via 572 edges. Figure 6 on the top gives a schematic illustration of the model. The metabolites within the PN were numbered. Their names are listed in the Supplementary file Table 1.pdf. The reactions, including the literature references, are compiled in the Tables 2–13 in the Supplementary file Table 1.pdf. The interface to the environment was modeled by 29 reactions indicated by an *IN* or *OUT* in the prefix of their names. The input reactions create the substrates glycine (metabolite = place 18), D-fructose (place 63), D-galactose (place 73), coenzyme A (CoA, place 93), acetyl-CoA (place 92), ammonia (place 29), or citrulline (place 94). In turn, the output reactions create 22 metabolites (see Table 14 in the Supplementary file Table 1.pdf). 18 output transitions were connected to sink metabolites.

Four metabolites were connected to both, an input and output transition, forming external metabolites (CoA, ammonia, acetyl-CoA, and citrulline). The biosynthesis of CoA was not modeled, and thus, the precursor of CoA, pantothenate (Raman and Rathinasabapathi, 2004), is not present in the PN. Similarly, acetyl-CoA is a product of the β-oxidation of fatty acids in *A. thaliana* (Fulda et al., 2002) which is not part of the model as well. Nitrate and a ammonia were taken up by roots and transported to photosynthetic tissues (Chalot et al., 2006). In leaves, ammonium is the primary nitrogen source for glutamine synthesis in the cytosol or chloroplasts, while nitrogen is either stored in vacuoles or converted to ammonium for further processing. For intracellular transport of ammonium between mitochondria and chloroplasts, a so-called ornithine-citrulline shuttle is proposed (Linka and Weber, 2005), which is also discussed to represent an efficient transport system for carbon dioxide from mitochondria to chloroplasts in form of citrulline. We modeled the external setting of citrulline to dissolve an occurring deadlock, consisting of the metabolites 94 (citrulline), 27 (L-arginino-succinate), 28 (arginine), and 1 (ornithine). Considering the four metabolites that serve as input and output, the PN consumed 3 input metabolites to produce 18 output metabolites.

For this complete PN, we were not able to compute the TIs. To verify the PN we first manually divided the complete PN into four smaller subnetworks of known biological meaning, for which we could separately compute the TIs. We chose four subnets of biological relevance according to the literature: (1) the sucrose (Figure 6, blue), (2) the citrate (red), (3) the shikimate (yellow), and (4) the UTP subnet (green). The names of the subnets represent the key metabolites of each subnet. The sucrose subnet was supplied with D-fructose and D-galactose as input from the environment, while the other five input metabolites were fed into the citrate subnet. In turn, nine metabolites were exported from the sucrose subnet, five from the citrate subnet, five from the shikimate subnet, and three from the UTP subnet.

#### 3.1.1. The sucrose subnet

**Figure 7:**
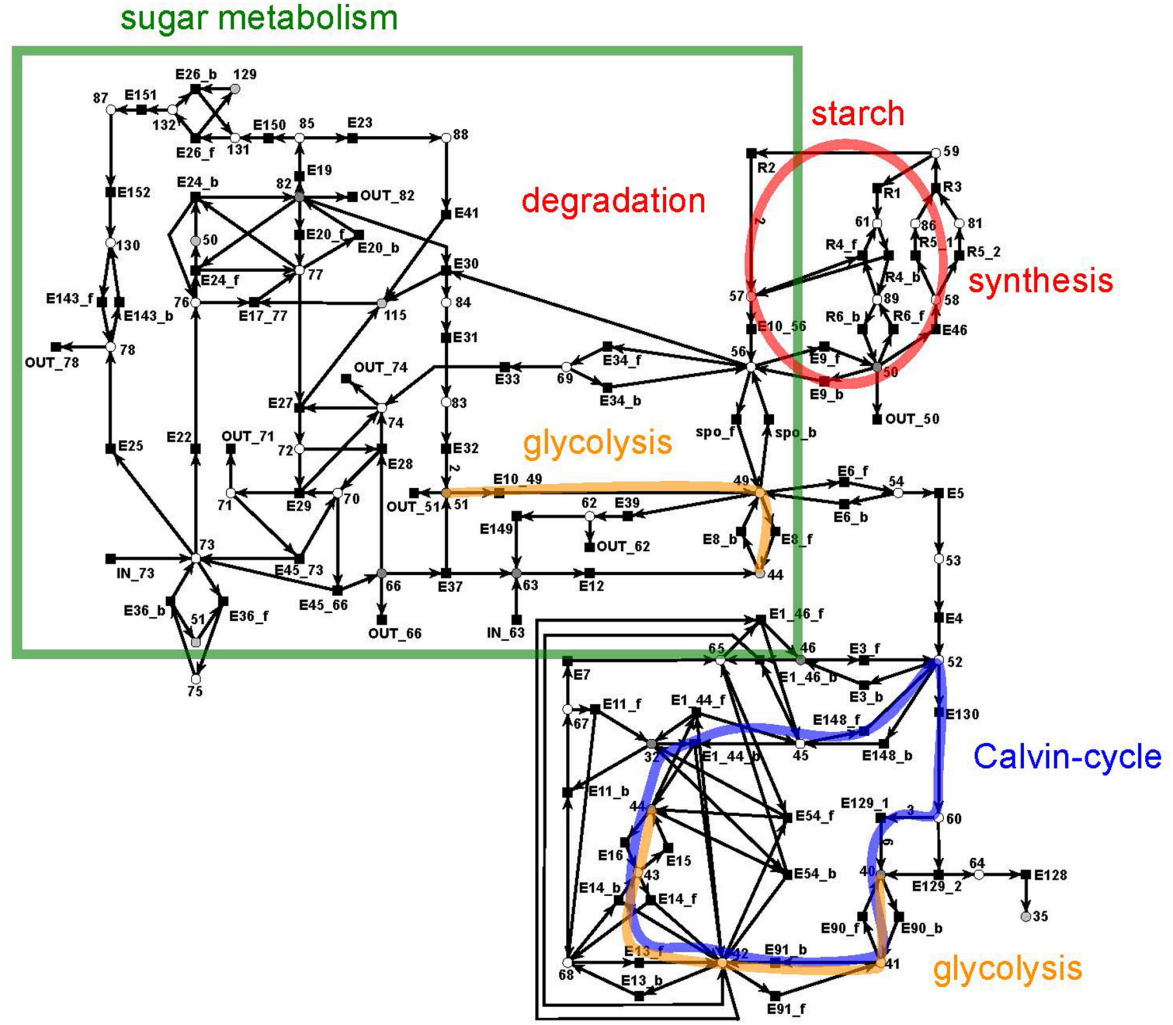
The PN model of the sucrose subnet adapted from Nöthen (2014). It covers the Calvin cycle (blue), part of the glycolysis (yellow), the sugar metabolism (green box), and the starch synthesis and degradation (red circle).

The sucrose subnet consists of 44 metabolites and 103 reactions (Figure 7). The subnet contains seven additional input reactions and 22 additional output reactions to generate a sufficient outlet of products. The metabolites involved in these reactions link the sucrose network with the other parts of the PN. 18 output transitions were connected to sink metabolites.

The sucrose subnet consists of four pathways, the Calvin cycle (Figure 7, blue), the sugar metabolism (red), the glycolysis (yellow), and the starch metabolism (green). The Calvin cycle is central for carbon fixation in plants, while the sugar metabolism accomplishes the synthesis and degradation of sucrose (metabolite 66) and UDP-glucose (metabolite 82). The glycolysis (yellow, orange) appeared to be disconnected in the layout, but the reactions of the glycolysis were combined via the logical place 44 (β-D-fructose 6-phosphate). However, only a part of the glycolysis pathway belongs to the sucrose network—the reaction cascade from D-glucose (metabolite 51) to glycerate 3-phosphate (metabolite 40). In turn, the other part of the glycolysis pathway is integrated in the citrate subnet. Thus, a transfer metabolite was required for the complementation of this cycle.

#### 3.1.2. The starch metabolism

**Figure 8:**
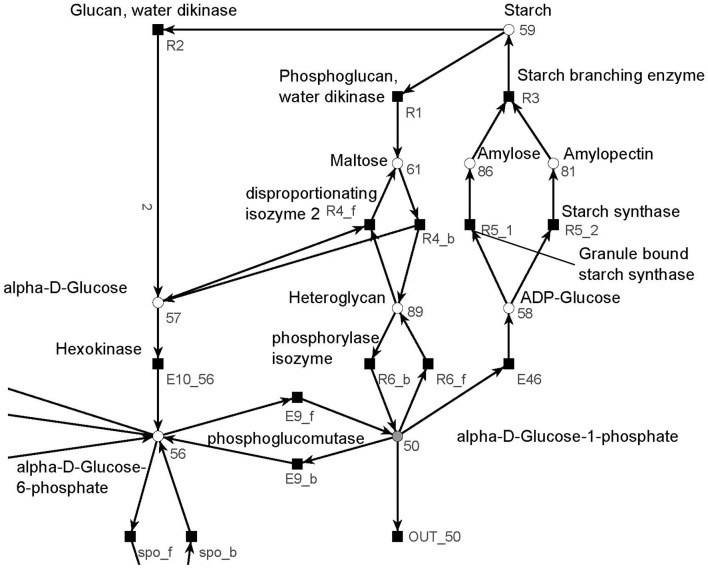
The PN model of the starch subnet adapted from Nöthen (2014). It covers the starch synthesis and degradation.

**Figure 9:**
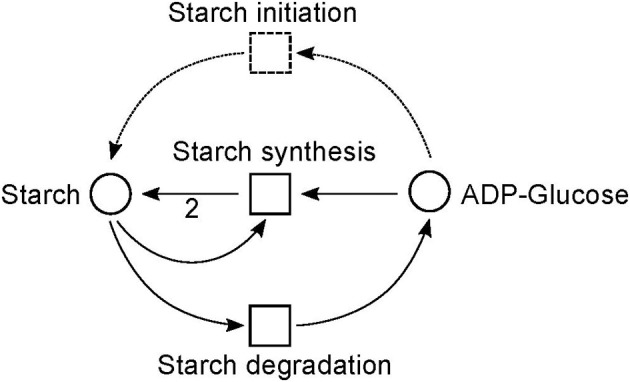
The simplified PN model of the initiation, synthesis, and degradation of starch adapted from Nöthen (2014). The PN is not pure, because the place *Starch* is a pre-place as well a post-place of the transition *Starch synthesis*. The starch initiation (dashed lines) was required to avoid a deadlock. In the presence of glucose, the metabolite starch is able to auto-catalyze the production of more starch until no starch is present in the system, e.g., all starch has been degraded. Without the starch initiation, the production of starch would be blocked, producing a deadlock.

The metabolism became obvious by inspecting the synthesis and degradation of starch in detail based on Figure 7 (red). The priming, chain-addition polymerization, polymer degradation, irreversible poly-condensation, and granule formation of starch are complex enzymatic processes. The mechanisms underlying these processes are not yet fully understood for *A. thaliana* (Szydlowski et al., 2009). While constructing the PN (Figure 8), we did not account for the diverse structures of starch macromolecules and for the chain length distribution. Thus, we lumped the diversity of starch macromolecules to a single unique metabolite named starch (metabolite 59). This simplification was supported by a biologically intuitive view of the coarse-grained structure of the net. We adopted previously presented suggestions (Kossmann and Lloyd, 2000; Guy et al., 2008; Fettke et al., 2009) to model the starch metabolism as a pure, deadlock-free PN. To indicate deviations of reactions from the activity of single enzymes we replaced the prefix *E* (for enzyme) by *R* (for reaction) in the names of such reactions. More precisely, in Figure 9, two molecules of ADP-glucose (metabolite 58) form one molecule of starch (metabolite 59) via amylose (metabolite 86) and amylopectin (metabolite 81) (Kossmann and Lloyd, 2000). The breakdown of one unit of starch produces two units of α-D-glucose (metabolite 57) or, alternatively, via maltose (metabolite 61) only one unit of α-D-glucose and one unit of heteroglycan (metabolite 89) (Fettke et al., 2009). Heteroglycan is freely convertible to α-D-glucose 1-phosphate (metabolite 50). Also α-D-glucose can be converted via α-D-glucose 6-phosphate (metabolite 56) to α-D-glucose 1-phosphate (metabolite 50). α-D-glucose 1-phosphate is represented by a logical place (gray-filled circle). Thus, the metabolite α-D-glucose 1-phosphate can be produced and consumed by several reactions also outside the sucrose subnet. The reaction, *E*46, transforms α-D-glucose 1-phosphate to ADP-glucose, which can be consumed for further production of starch.

Due to the polymer character of the starch molecule, it was difficult to model its synthesis and degradation in detail. There is literature (Kossmann and Lloyd, 2000; Lu and Sharkey, 2006; Guy et al., 2008; Reiter, 2008; Fettke et al., 2009) available that describes the reaction cascade of the starch metabolism without special focus on the polymeric structure of starch. We put special effort into the curation of the thermodynamic feasibility of the starch pathway by an adaption of the stoichiometric parameters in the cascade in such a way that no substance was created or consumed (comparable to the procedure in Poolman et al., 2009). This thermodynamic feasibility was supported by the two cyclic TIs, one for each degradation pathway, which together covered the starch metabolism.

#### 3.1.3. The citrate subnet

**Figure 10:**
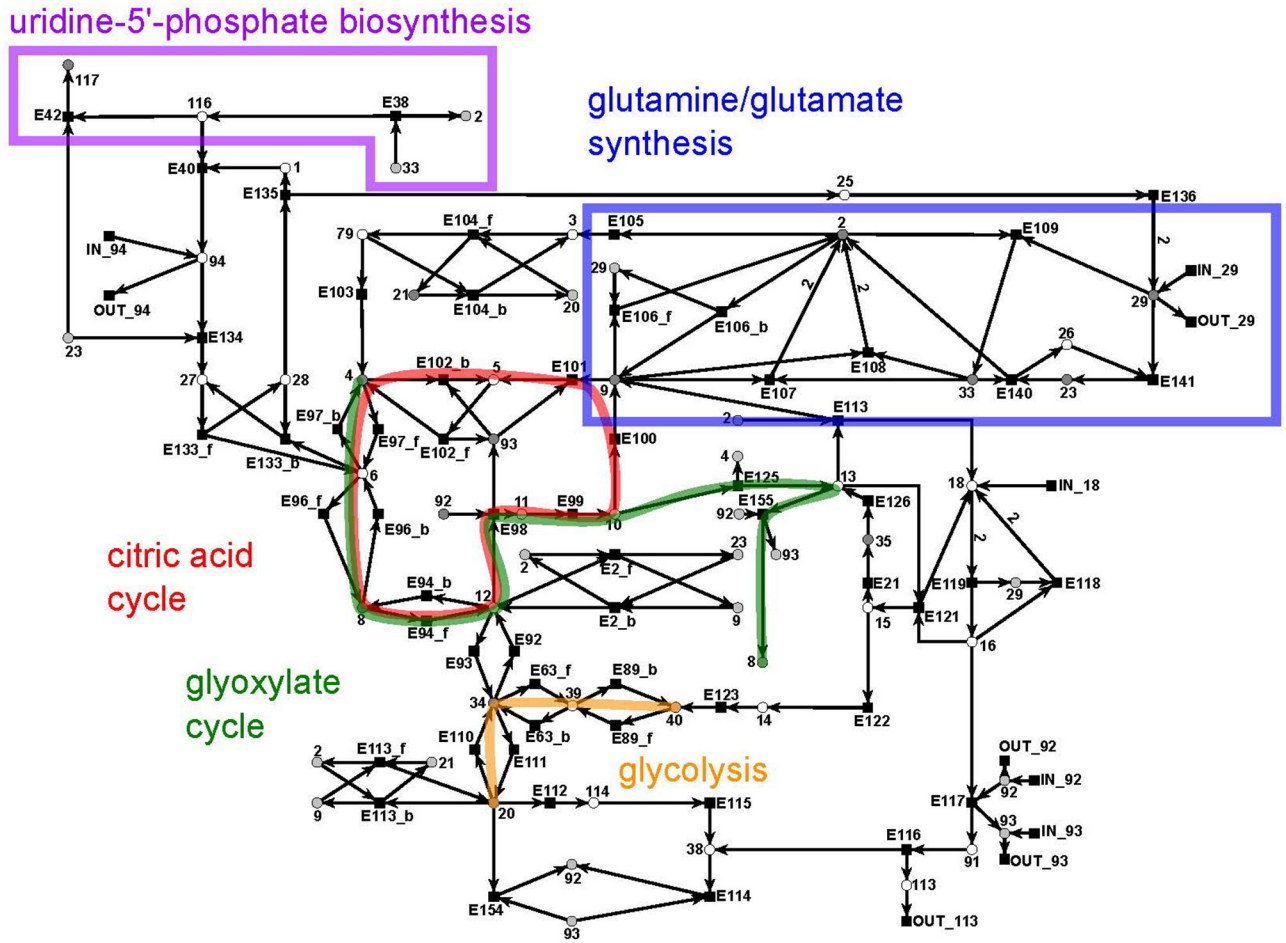
The PN model of the citrate subnet adapted from Nöthen (2014). It covers the citric acid cycle (red), the glyoxylate cycle (green), part of the biosynthesis of uridine 5′-phosphate (purple box), the biosynthesis of glutamate and glutamine (blue box), and part of the glycolysis (yellow).

The citrate subnet consists of 43 metabolites and 79 reactions (Figure 10). The subnet contains two additional input and five additional output reactions that connect the citrate network with the other parts of the PN.

This subnet specifies the biosynthesis of glutamate (metabolite 2) and glutamine (metabolite 33) (blue), the citric acid cycle (red), and the glyoxylate cycle (green), part of the biosynthesis of uridine 5′-phosphate (violet), and completes the pathway of glycolysis (yellow). Glutamine and glutamate play a central role in the transfer of ammonia (metabolite 29). The citric acid cycle is part of the energy metabolism in aerobic species. The glyoxylate cycle (glyoxylate: metabolite 13) plays a major role in the anabolic synthesis of carbohydrates. The reactions of glycolysis complement the part of the cycle integrated in the sucrose subnet by converting glycerate 3-phosphate (metabolite 40) to pyruvate (metabolite 20) (yellow). The biosynthesis of uridine 5′-phosphate (uridine mono phosphate, UMP) is only partially present in this subnet and was completed in the UTP subnet (purple).

#### 3.1.4. The shikimate subnet

**Figure 11:**
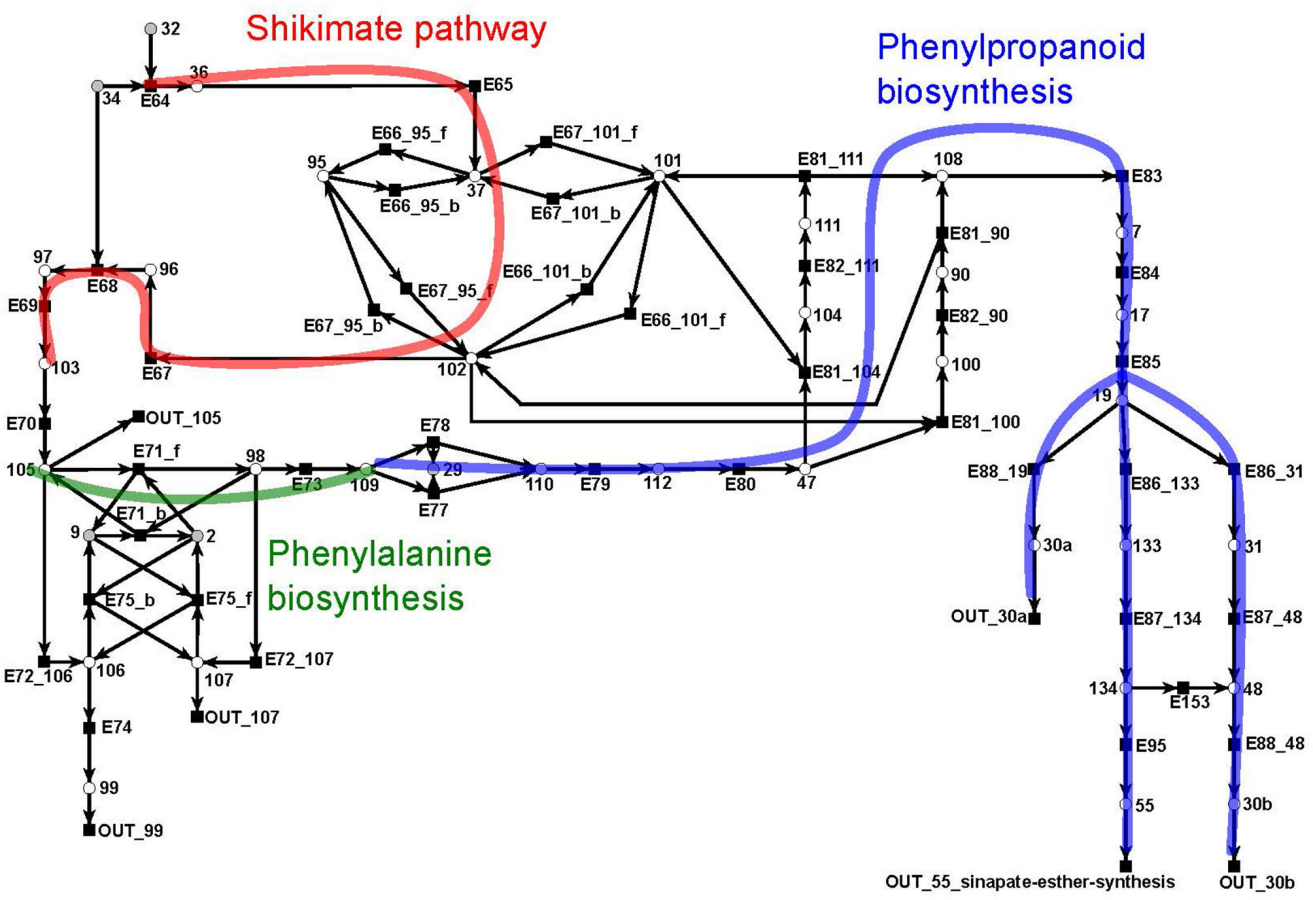
The PN model of the shikimate subnet adapted from Nöthen (2014). It covers the shikimate pathway (red), the biosynthesis of phenylalanine (green), and of phenylpropanoid (blue).

The shikimate pathway (Figure 11) consists of 39 metabolites and 46 reactions. It indicates the synthesis of shikimate (metabolite 102: red) as precursor for the synthesis of aromatic amino acids, e.g., phenylalanine (metabolite 109: green) (Herrmann and Weaver, 1999). In turn, shikimate and phenylalanine are precursors for phenylpropanoids (blue) which, in turn, are precursors for the lignin (metabolites 30*a* and 30*b*) synthesis. The synthesis of lignin is e.g., explicitly included as a part of the biomass function in a genome scale flux balance model of maize (*Zea mays*) (Saha et al., 2011). We divide lignin into two compounds, 30*a* and 30*b*, representing guaiacyl lignin and syringyl lignin, respectively. Guaiacyl lignin is believed to be synthesized from coniferyl alcohol (metabolite 19) while syringyl lignin is produced from sinapyl alcohol (metabolite 48) (Humphreys and Chapple, 2002).

#### 3.1.5. The UTP subnet

**Figure 12:**
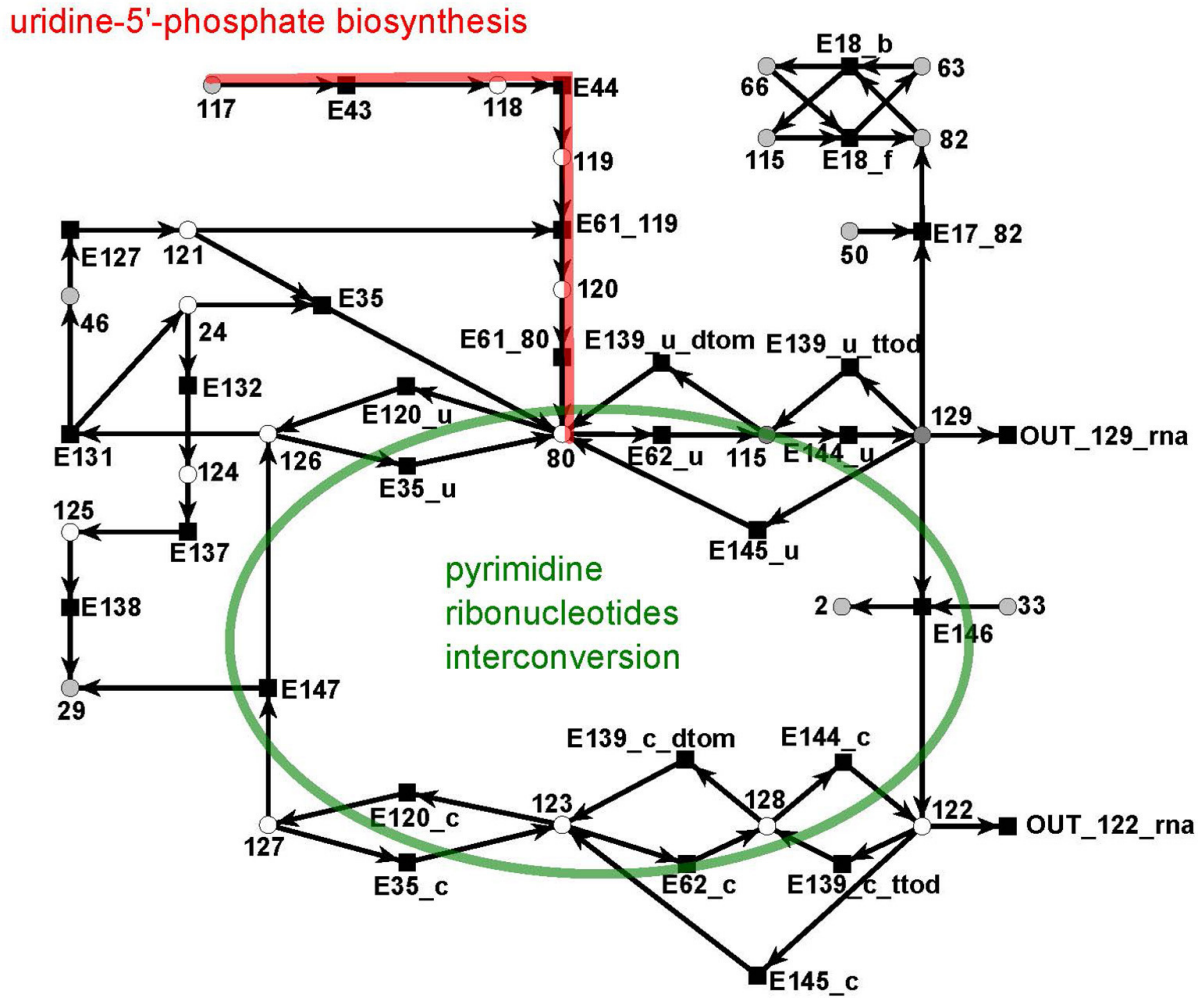
The PN model of the UTP subnet adapted from Nöthen (2014). It covers part of the synthesis of uridine 5′-phosphate, UMP, (red), and the interconversion of the pyrimidine ribonucleotides. The interconversion are the reversible transformation of UMPP, UDP, and UTP and CMP, CDP, and CTP into each other. Additionally, it contains the CTP synthesis CTP from UTP and degradation of CMP to UMP.

The UTP subnet is depicted in (Figure 12, red). It consists of 25 metabolites and 29 reactions. The connection with the other parts of the PN was achieved by three additional output reactions (Table 14 in the Supplementary file Table 1.pdf).

The subnet contains the completion of biosynthesis of UMP (Figure 12, red) that is initiated within the citrate subnet by consuming N-carbamoyl-L-phosphate (metabolite 117) to produce UMP (metabolite 80). The second major part of this subnet describes the interconversion of the pyrimidine ribonucleotides, i.e., the connection between UMP, UDP, and UTP and between CMP, CDP, and CTP, and the synthesis of CTP from UTP and the degradation of CMP to UMP (green).

### 3.2. The reduced petri net model

#### 3.2.1. Removal of metabolites

To limit the network complexity as far as possible, all small metabolites like water and carbon dioxide, catalytic substances like ions, and cofactors like NAD/NADH and the energy-providing compounds like ATP were omitted from the model. Hereby, the model was reduced by 19 metabolites and 274 edges. Studies have proven that hubs are less conserved than compounds which connect different modules (Guimera and Amaral, 2005), suggesting that a model without cofactors and small metabolites is more suitable than a model which lacks essential connections between the different biological modules. A complexity reduction by removing such smaller metabolites was preferred to a restriction of the overall network size. Several of the small metabolites, namely CoA and UDP, remained in the network due to suggestions of our coworkers (Schleiff, 2010, personal communication). To prove that the reduced network was still a real-world network, we compared the distributions of vertex degrees of the reduced network with that of the network based on the AraCyc database. After excluding all metabolites with a vertex degree greater than 74, we could show that the distribution of vertex degrees of the AraCyc network was very similar to the one of the PN model. This indicated that the reduced PN model was also a real-world model.

We applied ITPs (Invariant Transition Pairs) and CTPs (Common Transition Pairs) using an extended version of a previously published algorithm (Ackermann et al., 2012; Nöthen, 2014). We searched first for CTPs and then for ITPs in a parallel running implementation. If any of these structures were encountered, the net was reduced and followed by a search for a CTP. We considered the following cases: (1) there are CTPs that as well form ITPs. This is the case, if a place is connected to the rest of the network only via an ITP-forming transition pair. Then, the algorithm reduces the ITP first. (2) A special case of an ITP is given, if a certain metabolite is connected to an input and output transition. The PN model contains several external metabolites which fulfill these special ITP rules.

86 CTPs, 62 ITPs, and 2 parallel reduction steps were performed. All steps were listed in the supplementary file Table 2.pdf in the Tables 1–9. The number of metabolites was reduced from 134 to 60, the number of reactions from 243 to 131, and the number of edges from 572 to 329.

Figure 6 depicts in a schematic way the original PN model (on the top) and the model after reduction (on the bottom). We exemplarily demonstrated the interpretation of the results of the reduction procedure, choosing a place following from a series of ITP reductions. The place represented a conglomeration of substances involved in the Calvin cycle, the glycolysis, and the citric acid cycle. It combined the compounds succinate (metabolite 4), fumarate (metabolite 6), malate (metabolite 8), oxalacetate(metabolite 12), pyruvate (metabolite 20), phosphoenolpyruvate (metabolite 34), glycerate 2-phosphate (metabolite 39), glycerate 3-phosphate (metabolite 40), glycerate 1,3-bisphosphate (metabolite 41), glyceraldehyde 3-phosphate (metabolite 42), and dihydroxyacetone phosphate (metabolite 68) into the same place. These reduction steps additionally removed the transitions *E*13, *E*14, *E*15, *E*90, and *E*91, while the transitions *E*11 and *E*14 remained in the reduced PN. The first four metabolites were part of the citric acid cycle. Out of the remaining seven metabolites which were part of the glycolysis, there were five, namely glycerate 2-phosphate (metabolite 39), glycerate 3-phosphate (metabolite 40), glycerate 1,3-bisphosphate (metabolite 41), glyceraldehyde 3-phosphate (metabolite 42), and dihydroxyacetone phosphate (metabolite 68), which were also part of the Calvin cycle.

In plastids of *A. thaliana*, the glycolysis and the Calvin cycle shared several enzymes (Peltier et al., 2006): phosphogylcerate kinase (transition *E*90, reversible), glyceraldehyde 3-phosphatedehydrogenase (transition *E*91, reversible), triosephosphate isomerase (transition *E*13, reversible), seduheptulosebisphosphate aldolase (transitions *E*11 and *E*14, both reversible), and fructose bisphosphatase (transition *E*15, reversible by transition *E*16). The sharing of these reactions suggested that the participating compounds were shared as well. In this case, the shared metabolites would be: glycerate 3-phosphate (metabolite 40), glycerate 1,3-bisphosphate (metabolite 41), glyceraldehyde 3-phosphate (metabolite 42), D-fructose 1,6-bisphosphate (metabolite 43), D-fructose 6-phosphate (metabolite 44), and dihydroxyacetone phosphate (metabolite 68) besides D-fructose 1,6-bisphosphate (metabolite 43) and D-fructose 6-phosphate (metabolite 44). The absence of D-fructose 1,6-bisphosphate (metabolite 43) and D-fructose 6-phosphate (metabolite 44) in the reduced place was caused by restrictions in the reduction process. A merging of places by an ITP reduction was only allowed if all involved edges had a weight of 1. This was not the case for the reactions connecting metabolite 43 with 42 and 68 (transition *E*14). The merging of parts of the citric acid cycle, namely succinate (metabolite 4), fumarate (metabolite 6), malate (metabolite 8), and oxalacetate (metabolite 12), was inspired by the reversible reactions between the compounds, which was the main idea behind an ITP reduction. The combination of this part of the citric acid cycle and the last steps of the glycolysis, producing phosphoenolpyruvate and pyruvate, was induced by the synthesis pathway of oxalacetate from phosphoenolpyruvate (Dey and Harborne, 1997). Considering these biological aspects, the reduction process made sense.

Additionally, parallel structures of connections between two metabolites were merged as described in other PN analyses (Murata, 1989; Reddy et al., 1993). After 86 CTP and 62 ITP reduction steps, the resulting reduced network consists of 60 metabolites (45% of originally 134), 131 reactions (54% of originally 243), and 329 edges (58% of originally 572).

### 3.3. Transition invariant analysis

The problem of enumerating all minimal TIs can not be solved in polynomial time. The overall complexity of this task is still not clear, and the decision problem, whether two transitions occur in the same TI, is NP-complete (Acuña et al., 2010).

The complete model was too complex for the computation of all the TIs. Several weeks of computation time on an AMD *Opteron*^*T*^*M* 2.2 GHz with 32 GB RAM did not lead to any result. Therefore, we decomposed the PN into four biologically motivated subnetworks, which form two modules, the sucrose module, combining the sucrose and the UTP subnetwork, and the citrate module, combining the citrate and the shikimate subnet. Additionally, we reduced the complete network, preserving the CTI property. We computed the complete set of TIs for the two modules and for the reduced network. All these networks were covered by TIs. Table 1 compiles the number of TIs for the two modules, the reduced network, and for all TI types.

**Table 1:** The numbers of places, transitions, and TIs in the modules and in the reduced network.

**PN size**	**Sucrose module**	**Citrate module**	**Reduced PN**
Places	69	72	60
Transitions	132	125	131
**TI type**			
All	4,602	3,214	27,646
Trivial	31	25	22
IN + OUT	4,473	3,140	26,095
IN	18	41	1,298
OUT	63	0	132
Cyclic	17	8	99

*All networks were CTI. We grouped the TIs according to the type of interface to the environment. INOUT TIs contained input and output transitions, IN TIs only input transitions, and OUT TIs only output transitions. Cyclic TIs contained neither IN nor OUT TIs. Trivial invariants were reversible reactions which were split into a forward and a backward transition*.

The explanations for some of the different types of invariants were intuitive. In a metabolic PN, which usually contains reversible reactions, it was not possible to avoid trivial TIs. The condition of minimality ensures that no other invariant contains both of the transitions of the reversible reaction. TIs mainly represent pathways through the network, a succession of consecutive biochemical reactions, transforming given educts (metabolites produced by input transitions) to the corresponding products (metabolites consumed by output transitions). All TIs, containing OUT only, which exist in the sucrose module, could be explained easily.

We could show that a mapping of reduced invariants to invariants of the unreduced net will be possible.

The PN model did not include secondary metabolites, such that carbon dioxide was not modeled. In the Calvin cycle (Bassham et al., 1954; Calvin, 1956), carbon dioxide is bound to ribulose-1,5-biphosphate by ribulose-1,5-biphosphate carboxylase oxygenase (Parry et al., 2003; Raines, 2003; Roy and Andrews, 2004). This process produces two trioses from one pentose, thereby raising the number of carbons in the system. Due to deleted carbon dioxide, we modeled the Calvin cycle without an explicit input transition for carbon dioxide. All OUT TIs contained one of the two transitions modeled rubisco (*E*129_1 and *E*129_2) and thereby an implicit IN TI of carbon dioxide. This implicit modeling reduced all OUT TIs to INOUT pathways. Transition *E*129_1 modeled the carboxylation reaction (Calvin cycle) of rubisco, and transition *E*129_2 the oxygenation reaction (photorespiration) (Eckardt, 2005). All other modules did not possess OUT TIs. The OUT TIs of the reduced model were comparable to those of the sucrose subnet.

#### 3.3.1. An example for a cyclic transition invariant

**Figure 13:**
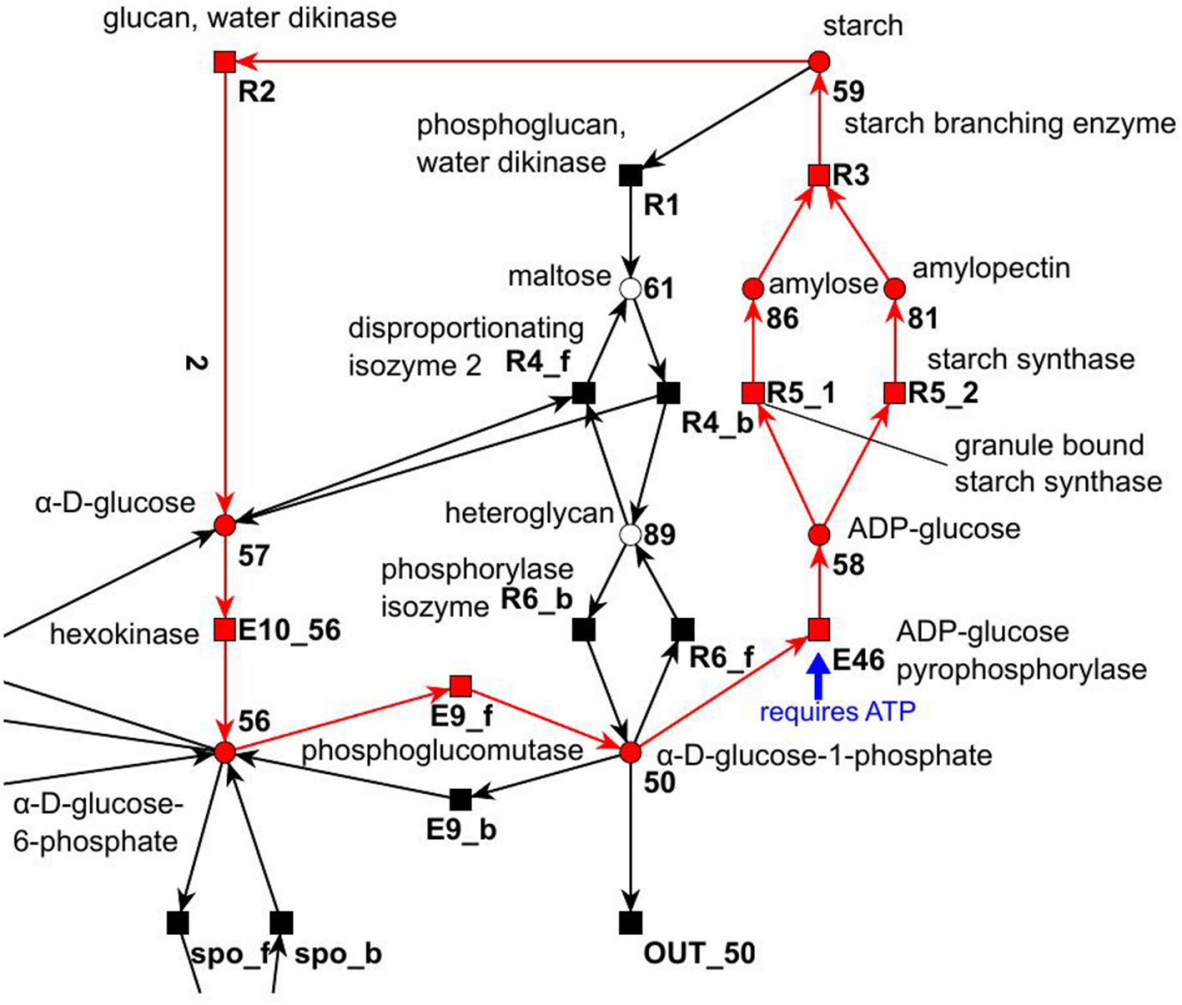
Example of a cyclic TI that describes part of the starch synthesis and degradation. The cycle evolves out of a silent input transition. The underlying reaction of transition *E*46 produces ADP-glucose and pyrophosphate from ATP and α-D-glucose 1-phosphate (Streb and Zeeman, 2012).

All cyclic TIs were as well artifacts, resulting from missing metabolites and cofactors. Figure 13 illustrates an example of a cyclic TI. The modeled starch metabolism requires ATP and α-D-glucose 1-phosphate (metabolite 50) to form ADP-Glucose (metabolite 58) and pyrophosphate by transition *E*46 (Kossmann and Lloyd, 2000; Streb and Zeeman, 2012). ATP and pyrophosphate were not modeled, otherwise this reaction would had required ATP and produced pyrophosphate. This led to two options, (1) ATP and pyrophosphate were directly provided and removed, and (2) ATP and pyrophosphate were produced and consumed throughout the network. Both cases resulted directly or indirectly in the involvement of input and output transitions leading to an INOUT TI.

IN TIs were as well caused by substances, which were not modeled. In the sucrose subnet, all IN TIs were artifacts of the modeling of the pentose phosphate pathway. Transition *E*4 produces a carbon dioxide (Kruger and von Schaewen, 2003) in the pentose phosphate pathway from metabolite 53 (6-phospho gluconate). As carbon dioxide was not modeled, this had the function of a hidden export. This behavior corresponded to the OUT TIs, which were artifacts of the missing modeling of carbon dioxide as well.

#### 3.3.2. Exemplary inspection of a single transition invariant

Here, we want to illustrate an exemplary inspection of a single TI of the reduced model, consisting of twelve reactions. As the reduction process merges reactions, the number of traversed reactions in the original PN could be higher. Table 2 illustrated the considered exemplary TI and listed the syntheses of substrates a reaction was involved in. Several biological pathways could be combined to form a single TI (Koch et al., 2005). The considered TI produced UTP for the synthesis of RNA from D-fructose (metabolite 63) and ammonia (metabolite 29). To biologically verify this pathway combination, we had to demonstrate that each part of the TI could be biologically explained. The product of the TI was two UTPs, which was removed from the network by the transition *OUT*_129_*rna*. The used compounds for this product were three D-fructoses provided by *IN*_63 and four ammonia provided by *IN*_29. In plants, one UMP was synthesized from four different compounds: one bicarbonate, one glutamine, one 5-phosphoribosyl 1-pyrophosphate, and one L-aspartate (Zrenner et al., 2006). UTP was then synthesized from UMP by adding phosphor groups. The metabolite bicarbonate was not modeled in the PN. It was demonstrated in the following that each of the three remaining modeled compounds was synthesized and used in a biologically explainable way. Two L-aspartate were synthesized by firing of transition *E*2_*f* (aspartate aminotransferase) two times. The substrates of this reaction were oxalacetate and glutamate and the products L-aspartate and α-ketoglutarate (Wilkie and Warren, 1998; Graindorge et al., 2010). In the reduction process, several pathways, leading to oxalacetate, were affected, resulting in a merged metabolite, which represents various compounds. The affected pathways were the glycerate 3-phosphate synthesis, the oxalacetate synthesis, and the glycolysis.

**Table 2:** Exemplary TI in the reduced model.

**Reaction and its firing frequency**	**Involved in the synthesis of**
2^*^E2_*f*	L-aspartate
*E*14_*f*	L-aspartate
3^*^IN_63	L-aspartate, 2^*^5-phosphoribosyl 1-pyrophosphate
3^*^E12	L-aspartate, 2^*^5-phosphoribosyl 1-pyrophosphate
2^*^ctp(*E*5 + *E*4)	5-phosphoribosyl 1-pyrophosphate
2^*^E127	5-phosphoribosyl 1-pyrophosphate
4^*^IN_29	2^*^ glutamate, 2^*^ glutamine
2^*^E106_*f*	Glutamate
2^*^E109	Glutamine
2^*^E38	Uridine 5–phosphate (UMP)
2^*^ctp(*ctp*(*E*42 + *E*43)+	Uridine 5–phosphate (UMP)
*ctp*(*E*44 + *ctp*(*E*61_119 + *E*61_80)))	
2^*^OUT_129_*rna*	RNA

*Each reaction was listed, including the number of its occurrences in the TI. Additional information was given about the syntheses the reaction was involved in, either the synthesis of one of the substrates L-aspartate, 5-phosphoribosyl 1-pyrophosphate, glutamine, and UMP, or the final synthesis of RNA. If different subpathways were assigned to the same reaction, the respective multiplicity was mentioned (see IN63 and E12)*.

**Figure 14:**
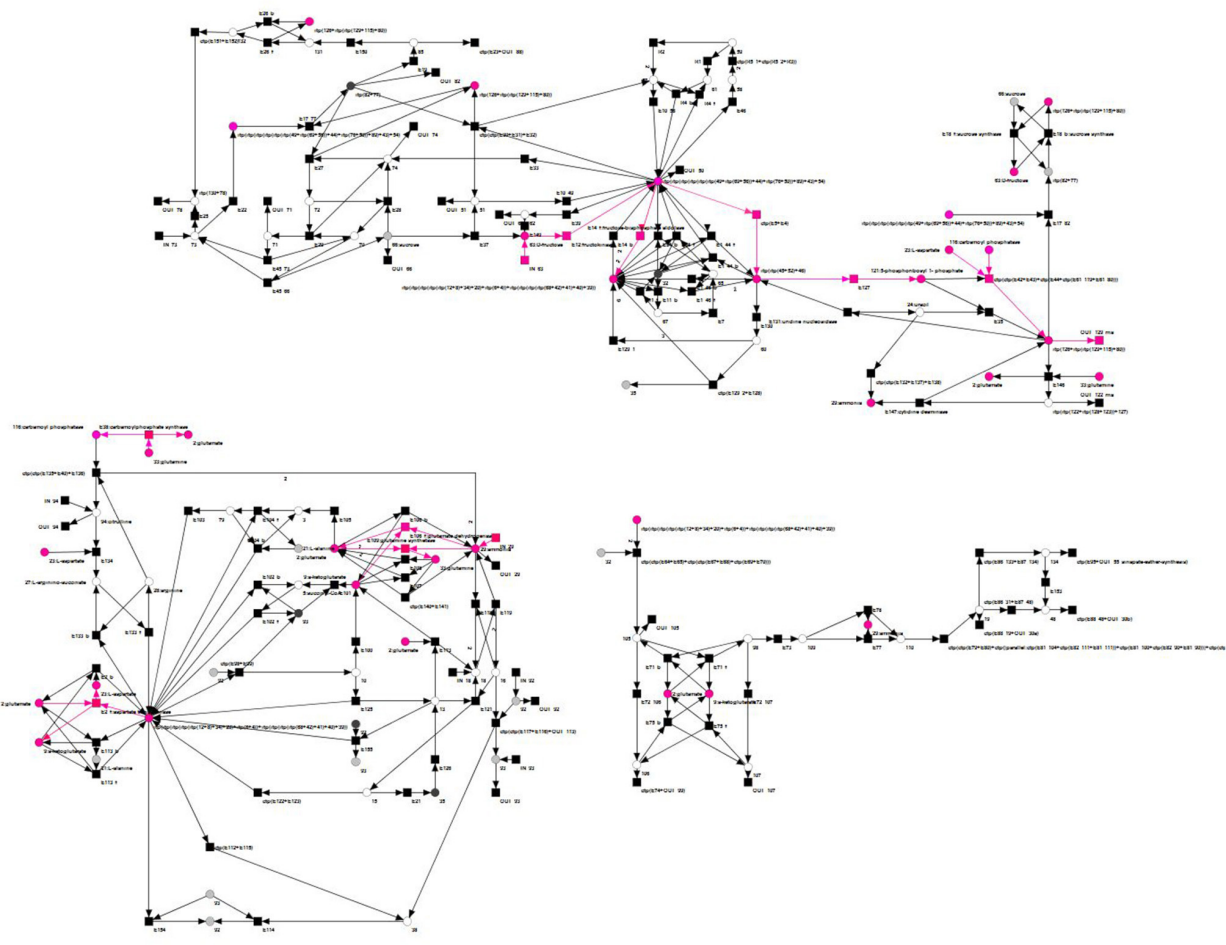
Overview of an examplary TI (pink) of the reduced PN that expresses a combination of parts of the nitrogen economy, the oxalacetate synthesis, the synthesis of 5-phosphoribosyl 1-pyrophosphate, and the synthesis of pyrimidines.

**Figure 15:**
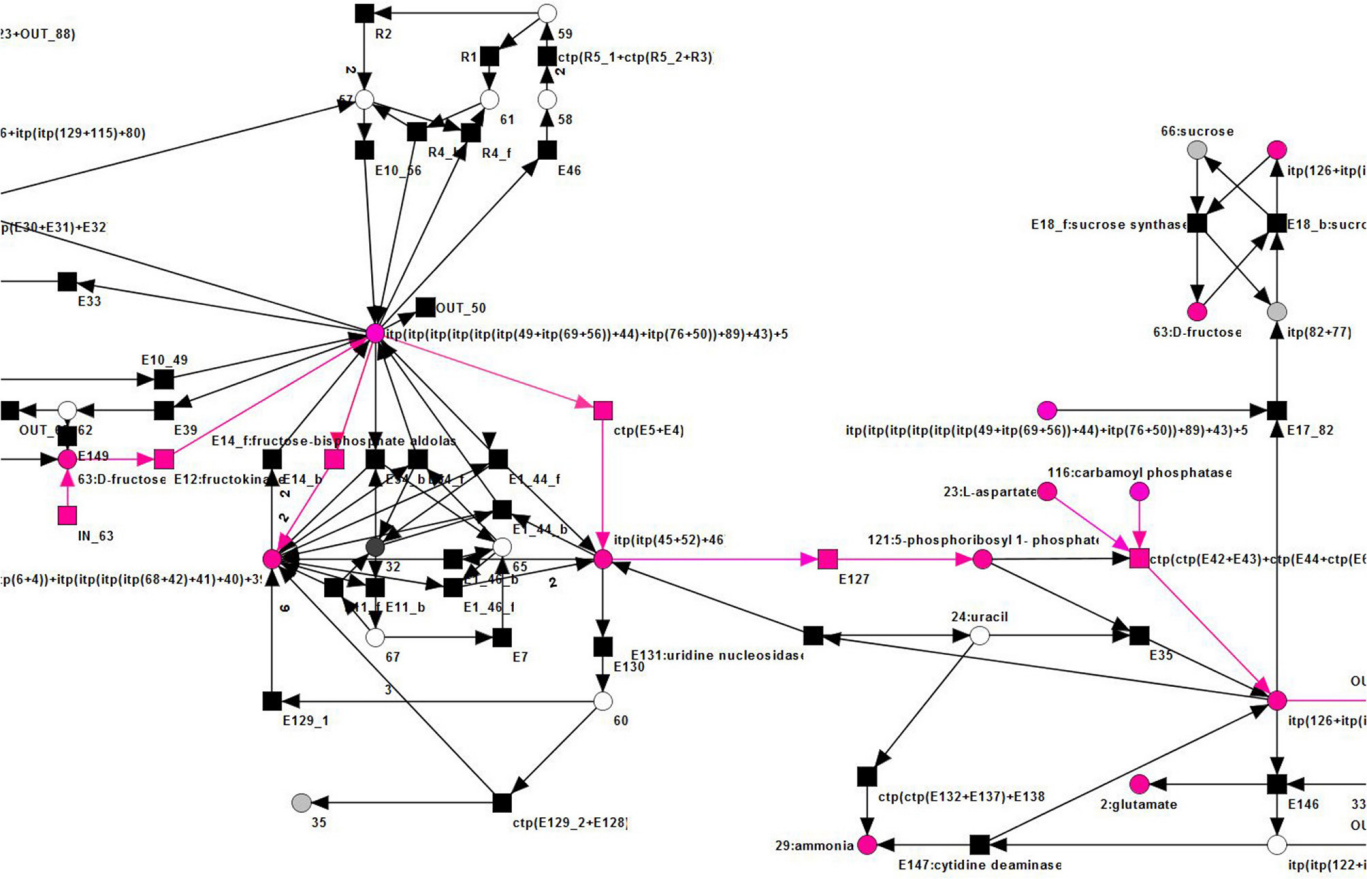
The top right part of an examplary TI (pink) of the reduced PN, see Figure 14.

5-phosphoribosyl 1-pyrophosphate can be synthesized from D-fructose, via D-fructose 6-phosphate and D-ribose 5-phosphate (Dey and Harborne, 1997; Buchanan et al., 2000; Berg et al., 2002; Zrenner et al., 2006). This pathway structure was represented in the TI by the reactions *E*12 (fructokinase, synthesis of D-fructose 6-phosphate), *ctp*(*E*5 + *E*4) (6-phosphogluconolactonase and phosphogluconate dehydrogenase, synthesis of D-ribose 5-phosphate), and *E*127 (5-phosphoribosyl 1-pyrophosphatesynthase, synthesis of 5-phosphoribosyl 1-pyrophosphate). Please note that a number of reactions in the overall synthesis of 5-phosphoribosyl 1-pyrophosphate was affected by the reduction process, and the precursor of transition *E*127 combined several compounds. The remaining reactions, *E*106_*f* and *E*109, were required to regenerate two glutamate and two glutamine, thereby using four ammonia. Glutamine is an important part of the nitrogen metabolism of conifers (Cánovas et al., 2007), and α-ketoglutarate is a known nitrogen transporter in plants (Temple et al., 1998). As glutamate is convertible in both, glutamine and α-ketoglutarate (Aubert et al., 2001; Forde and Lea, 2007), it seems to share these nitrogen-transportation duties. In the TI, the regeneration of glutamate and glutamine, respectively, from α-ketoglutarate and glutamate and in the process, consuming ammonia, resembled this biological interpretation, see Figures 14, 15. These findings proved this TI to be a combination of parts of the nitrogen economy, the oxalacetate synthesis, the synthesis of 5-phosphoribosyl 1-pyrophosphate, and the synthesis of pyrimidines. Altogether, these were the important steps to synthesize UTP (Zrenner et al., 2006). Each part of the inspected TI could be biologically interpreted, which proved the possibility of the network to model these syntheses in a steady-state sustaining manner.

### 3.4. MCT-sets

MCT-sets represent the smallest biologically meaningful entities in which a network can be decomposed (Sackmann et al., 2006). We give several examples of MCT-sets of the reduced PN model and their biological counterparts. This additionally provides a possibility of comparison between the biologically motivated subnets and the reduced network.

#### 3.4.1. MCTS 1 and the sucrose module

This MCTS consists of the transitions *Import*117*fromB*/*fromCit*, *E*43, *E*44, *E*61_119, and *E*61_80. All these transitions formed the reaction chain that leads to uridine 5′-phosphate (Zrenner et al., 2006). The import transition added carbamoyl aspartate to the modules, which was produced by transition *E*42 in the citrate module. *De novo* synthesis of uridine 5′-phosphate is highly energy consuming and in some tissues partly replaced by the recycling of already built compounds. Nevertheless, *de novo* synthesis of UMP is still needed to replenish the nucleotide stock (Moffatt and Ashihara, 2002). In the reduced network, this complete synthesis pathway was merged into one transition, called *ctp*(*ctp*(*E*42 + *E*43) + *ctp*(*E*44 + *ctp*(*E*61_119 + *E*61_80))). In the recursive reduction process, all of the reactions, forming the *de novo* synthesis of uridine 5–phosphate, fulfilled the conditions necessary for a CTP reduction. As transition *E*42 was part of the citrate module it could not be part of this MCTS. Nevertheless, the possible CTP reduction of the complete reaction chain, including *E*42, suggested a strong connection between the reactions of the uridine 5′ phosphate *de novo* synthesis and could indicate an MCTS in the PN, covering all of them.

#### 3.4.2. MCTS 2 and the citrate module

MCTS 2 consists of the transitions *E*103, *E*104_*f, E*105, and *E*113_*f*. This configuration occurred in the citrate module of the biologically driven decomposition as well as in the reduced network. Compared to the decompositions and the original network, the connections of the transitions have changed during the reduction process. In the subnets, the transitions form the reactions:

- $E103:79\overset{E103}{\to }4$
- $E104\_f:3+20\overset{E104\_f}{\to }21+79$
- $E105:2\overset{E105}{\to }3$
- $E113\_f:9+21\overset{E113\_f}{\to }20+2$

with 2 = glutamate, 3 = γ-aminobutyric acid (GABA), 4 = succinate, 9 = α-ketoglutarate, 20 = pyruvate, 21 = phosphoenolpyruvate, and 79 = succinate semialdehyde. In the reduced network, the metabolites 4 and 20 were combined by an ITP reduction, forming a new place. The connections of this new place were the combined connections of the merged original places, i.e., the new place connected to *E*103 (as metabolite 4), *E*104_*f* (as metabolite 20), and *E*113_*f* (as metabolite 20) and led to the reduced reaction system

- $E103:79\overset{E103}{\to }X$
- $E104\_f:3+X\overset{E104\_f}{\to }21+79$
- $E105:2\overset{E105}{\to }3$
- $E113\_f:9+21E113\_f\overset{E113\_f}{\to }X+2$

with 2 = glutamate, 3 = γ-aminobutyric acid (GABA), 9 = α-ketoglutarate, 21 = phosphoenolpyruvate, 79 = succinate semialdehyde, and *X* = the merged place (4 + 20). While the pathways for these transitions mentioned in the AraCyc database are glutamate degradation (*E*10, *E*104_*f*, and *E*105), and alanine degradation (*E*113*f*), other literature declare them as the GABA shunt (Bouché and Fromm, 2004). Finding an MCTS of these reactions strongly suggested a close interaction between them, indicating a possible network behavior consistent with the literature, because GABA is sufficient as sole nitrogen source for effective growth of *A. thaliana* (Breitkreuz et al., 1999), and the GABA shunt seems to play an important role in the reaction to oxidative stress (Bouché et al., 2003; Bouché and Fromm, 2004).

#### 3.4.3. MCTS 3 and the citrate module

This MCTS constitutes of the transitions *E*40, *E*133_*f, E*134, *E*135, and *E*136 in the citrate module. The transitions *E*40, *E*133_*f, E*134, and *E*135 formed the urea cycle (Tischner et al., 2007), and transition *E*136 modeled the degradation of urea (metabolite 25, Sirko and Brodzik, 2000). This MCTS is a collection of transitions forming and degrading urea. In the reduced network, two CTP reductions took place in this cycle. Initially, *E*135 and *E*40 were condensed to *ctp*(*E*135 + *E*40), which was further flattened to *ctp*(*ctp*(*E*135 + *E*40) + *E*136) by the inclusion of *E*136. Together with *E*134 and *E*133_*f*, this new reduced transition formed an MCTS in the reduced network. Urea is an important nitrogen source for plants (Polacco and Holland, 1993) and mainly believed to be predominantly synthesized by the urea cycle (Reinbothe and Mothes, 1962). This MCTS suggested the importance of the urea metabolism in the PN model of the core metabolism of *A. thaliana*.

## 4. Conclusion

In this paper, we presented a new semi-quantitative Petri net model of the metabolism of *A. thaliana* based on recent literature. Similar to a network of barley (Grafahrend-Belau et al., 2009), the model was manually developed and curated. To ensure the model's consistency, we used PN-based reduction and biologically motivated as well as graph-based decomposition and analysis techniques.

The final size of the complete PN model was 134 metabolites, 243 reactions, and 572 edges. The complexity of this model did not allow to compute all its transition invariants, which form the base for further analysis. To get a manageable set of TIs, we followed two strategies. First, we divided the model into four biology-driven subnetworks, the sucrose, the citrate, the UTP, and the shikimate subnetwork, and defined two modules, each consisting of two subnetworks. The sucrose module covers the sucrose and the UTP subnetwork, while the citrate module compiles the citrate and the shikimate subnetwork. The second strategy followed a graph-theoretic reduction of the model, applying common transition pairs and invariant transition pairs. Through the reduction, the network size decreased by ~50%. For all three subnetworks, we computed the TIs, easily showing that the subnetworks were CTI. To handle the amount of 27,646 TIs for the reduced model, we classified the TIs into trivial, INOUT, IN, OUT, and cyclic TIs. Because we could not discuss all the 27,646 TIs, we considered exemplarily one cyclic TI that describes a part of the starch synthesis and degradation and one TI that expresses a combination of parts of the nitrogen economy, the oxalacetate synthesis, the synthesis of 5-phosphoribosyl 1-pyrophosphate, and the synthesis of pyrimidines.

We demonstrated that the carbon fixation phase and the regeneration phase of the Calvin cycle strongly depends on each other. Additionally, potential steady-state pathways exist, which provided the fixed carbon to nearly all parts of the network, especially to the citric acid cycle. Moreover, the analysis showed a close cooperation of important metabolic pathways, e.g., the *de novo* synthesis of uridine-5–monophosphate, the γ-aminobutyric acid shunt, and the urea cycle.

The presented model provides a solid basis for further refinement, for example, by concentrations, gene expression data, and kinetic data for a quantitative analysis.

## Author contributions

ES and IK initiated and supervised the project. JN has performed the network construction and analysis. All authors wrote the manuscript.

### Conflict of interest statement

The authors declare that the research was conducted in the absence of any commercial or financial relationships that could be construed as a potential conflict of interest. The reviewer GR and handling editor declared their shared affiliation, and the handling editor states that the process nevertheless met the standards of a fair and objective review.
