# Supplementary material for: Modeling the Metabolism of Arabidopsis thaliana: Application of Network Decomposition and Network Reduction in the Context of Petri Nets
Source: Front Genet. 2017 Jun 30;8:85. doi: 10.3389/fgene.2017.00085 (PMC5491931; doi:10.3389/fgene.2017.00085)
Supplement: Supplementary file 1 [file Table1.PDF]

Supplementary Material: Article TitleA verified Petri model for the metabolism of *Arabidopsis thaliana*: application of network decomposition and network reduction

Ina Koch*[1], Joachim Nöthen[1] and Enrico Schleiff, [1] Shared first authorship

*Correspondence:
Ina Koch, Enrico Schleiff
ina.koch@bioinformatik.uni-frankfurt.de, schleiff@bio.uni-frankfurt.de

First Author 2: joachim.noethen@gmx.de

1 SUPPLEMENTARY TABLES

Supplementary Table 1. Metabolites of the complete PN model. *Name* corresponds to the metabolite's name. *No.* gives the number of the corresponding place.

No.	Name	No.	Name	No.	Name
1	ornithine	46	D-ribose 5-phosphate	91	acetyl-L-serine
2	glutamate	47	coumaroyl-CoA	92	acetyl-CoA
3	γ-aminobutyric acid (GABA)	48	sinapyl alcohol	93	coenzyme A (CoA)
4	succinate	49	β-D-glucose 6-phosphate	94	citrulline
5	succinyl-CoA	50	α-D-glucose 1-phosphate	95	3-dehydroshikimate
6	fumarate	51	β-D-glucose	96	shikimate 3-phosphate
7	feruloyl-CoA	52	D-ribulose 5-phosphate	97	5-enolpyruvyl-shikimate-3-phosphate
8	malate	53	6-phosphogluconate	98	L-arogenate
9	α-ketoglutarate)	54	D-glucono-1,5-lactone 6-phosphate	99	homogentisate
10	isocitrate	55	Sinapate	100	coumaroyl shikimic acid
11	citrate	56	α-D-glucose 6-phosphate	101	quinate
12	oxalacetate	57	α-D-glucose	102	shikimate
13	glyoxylate	58	ADP-glucose	103	chorismate
14	glycerate	59	starch	104	coumaroyl quinic acid
15	hydroxypyruvate	60	D-ribulose 1,5-bisphosphate	105	prephenate
16	serine	61	maltose	106	4-hydroxy-phenylpyrovate
17	coniferyl aldehyde	62	sorbitol	107	tyrosine
18	glycine	63	D-fructose	108	caffeoyl-CoA
19	coniferyl alcohol	64	2-phospho glycolate	109	phenylalanine
20	pyruvate	65	sedoheptulose 7-phosphate	110	trans-cinnamate
21	L-alanine	66	sucrose	111	caffeoyl quinic acid
22	not modeled	67	sedoheptulose 1,7-biphosphate	112	p-coumaric acid
23	L-aspartate	68	Dihydroxyaceton phosphate	113	cysteine
24	uracil	69	1L-myo-inositol-1-phosphate	114	acetaldehyde
25	urea	70	raffinose	115	uridine diphosphate (UDP)
26	asparagine	71	stachyose	116	carbamoyl phosphate
27	L-arginino-succinate	72	galactinol	117	carbamoyl aspartate
28	arginine	73	D-galactose	118	dihydroorotate
29	ammonia	74	D-myo-inositol	119	orotic acid
30a,b	lignin	75	galactane	120	orotidine 5'-monophosphate
31	hydroxyconiferyl alcohol	76	α-D-Galactose 1-phosphate	121	5-phosphoribosyl 1-phosphate
32	D-erythrose 4-phosphate	77	UDP-galactose	122	cytidine triphosphate
33	glutamine	78	L-galactono 1,4-lactone	123	cytidine monophosphate (CMP)
34	phosphoenolpyruvate	79	succinate semialdehyde	124	dihydrouracil
35	glycolate	80	uridine 5'-phosphate (UMP)	125	β-ureidopropionate
36	7-phospho-2-dehydro-3-deoxy-D-arabinoheptonate	81	amylopectin	126	uridine
37	3-dehydroquinate	82	UDP-glucose	127	cytidine
38	acetate	83	trehalose	128	cytidine diphosphate (CDP)
39	glycerate 2-phosphate	84	trehalose 6-phosphate	129	uridine triphosphate (UTP)
40	glycerate 3-phosphate	85	UDP-D-glucuronate	130	L-galactonate
41	glycerate 1,3-biphosphosphate	86	amylose	131	UDP-D-Galacturonate
42	glyceraldehyde 3-phosphate	87	D-galacturonate	132	D-galacturonate 1-phosphate
43	β-D-fructose 1,6-phosphate	88	UDP-xylose	133	hydroxyconiferyl aldehyde
44	β-D-fructose 6-phosphate	89	heteroglycan		
45	D-xylulose 5-phosphate	90	caffeoyl shikimic acid		

Supplementary Table 2. Reactions of the complete PN model. The column *Transitions* gives the reaction names. The reactions are named a combination of letters and numbers. A reaction name is represented by Ex, where x stands for an integer, which is random. Forward and backward reactions are indicated by Ex_f and Ex_f, respectively. IN_x stands for input reactions, where x denotes the supplied metabolite, and OUT_x for an output input reactions, where x denotes the removed metabolite. *Biochemical Pathway* denotes the biochemical pathways the reaction is participating. *Enzyme name* gives the name of the enzyme catalyzing the reaction. *References* refers to the publications where the reaction is described. *Reactions* describes the reactions' formulae and adds comments for special treatments. The metaboilites' numbers are encoded in Table 1.

Transition	Biochemical pathway	Enzyme name	Reference	Reactions
E1_44_b<br>E1_44_f<br><br>E1_46_b<br><br>E1_46_f	1. ribose degradation<br>2. pentose phosphate pathway (non-oxidative branch)<br>3. Calvin-Benson-Bassham cycle<br>4. Rubisco shunt	transketolase	Buchanan et al. (2000)	1. $45 + 46 \rightarrow 42 + 65$<br>2. $45 + 46 \rightarrow 42 + 65$&<br>$32 + 45 \rightarrow 42 + 44$<br>3. $42 + 65 \rightarrow 45 + 46$<br><br>4. $42 + 44 \rightarrow 32 + 45$&<br>$42 + 65 \rightarrow 45 + 46$
E2_f<br>E2_b	aspartate biosynthesis	aspartate transaminase	Wilkie and Warren (1998)<br>Graindorge et al. (2010)	$2 + 12 \rightarrow 23 + 9$<br>$23 + 9 \rightarrow 2 + 12$
E3_f<br><br>E3_b	1. ribose degradation<br><br>2. pentose phosphate pathway (non-oxidative branch)<br>3. Calvin-Benson-Bassham cycle<br>4. Rubisco shunt	ribose-5-phosphate isomerase	Berg et al. (2002)<br>Buchanan et al. (2000)	1. $46 \rightarrow 52$<br><br>2. $52 \rightarrow 46$<br><br>3. $46 \rightarrow 52$<br><br>4. $46 \rightarrow 52$
E4	pentose phosphate pathway<br><br>(oxidative branch)	phosphogluconate dehydrogenase	Dey and Harborne (1997)	$53 \rightarrow 52$
E5	pentose phosphate pathway (oxidative branch)	6-phosphoglu-conolactonase	Dey and Harborne (1997)	$54 \rightarrow 53$
E6_f<br>E6_b	pentose phosphate pathway (oxidative branch)	glucose-6-phosphat 1-dehydrogenase	Dey and Harborne (1997)	$49 \rightarrow 54$<br>$54 \rightarrow 49$
E7	Calvin-Benson-Bassham cycle	sedoheptulose-bisphosphatase	Lefebvre et al. (2005)	$67 \rightarrow 65$
E8	1. starch biosynthesis<br>2. gluconeogenesis<br>3. sucrose degradation III<br>4. glycolysis I (plastidic)	spontaneous	Nelson and Cox (2000)	1. $49 \rightarrow 56$<br>2. $56 \rightarrow 49$<br>3. $49 \rightarrow\leftarrow 56$<br>4. $49 \rightarrow\leftarrow 56$
E9_b<br>E9_f	1. starch degradation<br>2. starch biosynthesis<br>3. sucrose biosynthesis<br>4. UDP-glucose biosynthesis (from glucose 6-phosphate)<br>5. sucrose degradation III<br>6. superpathway of sucrose and starch metabolism I (non-photosynthetic tissue)	phosphogluco-mutase	Reiter (2008)	1. $50 \rightarrow 56$<br>2. $56 \rightarrow 50$<br>3. $56 \rightarrow 50$<br>4. $56 \rightarrow 50$<br><br>5. $50 \rightarrow 56$<br>6. $50 \rightarrow 56$
E10_56<br>E10_49	1. starch degradation<br>2. sucrose degradation III	hexokinase/ glucokinase	Lu and Sharkey (2006)<br>Guy et al. (2008)	$57 \rightarrow 56$<br>$51 \rightarrow 49$

Supplementary Table 3. Reactions of the complete PN model. For the legend see Table 2.

Transition	Biochemical pathway	Enzyme name	Reference	Reactions
E11_b<br>E11_f	Calvin-Benson-Bassham cycle	aldolase	Buchanan et al. (2000)	$32 + 68 \rightarrow 67$<br>$67 \rightarrow 32 + 68$
E12	sucrose degradation III	fructokinase	Guy et al. (2008)	$63 \rightarrow 44$
E13_b<br>E13_f	1. glycolysis IV (plant cytosol)<br>2. Calvin-Benson-Bassham cycle<br>3. glycolysis I (plastidic)	triose-phosphate isomerase	Berg et al. (2002)	$42 \rightarrow 68$<br>$68 \rightarrow 42$
E14_f<br>E14_b	1. Gluconeogenesis<br>2. Calvin-Benson-Bassham cycle<br>3. glycolysis I (plastidic)	fructose-bisphosphate aldolase	Guy et al. (2008)	$43 \rightarrow 42 + 68$<br>$44 + 68 \rightarrow 43$
E15	1. Gluconeogenesis<br>2. Calvin-Benson-Bassham cycle<br>3. glycolysis I (plastidic)	fructose-1,6-bisphosphatase	Guy et al. (2008)	$43 \rightarrow 44$
E16	1. glycolysis I (plastidic)<br>2. glycolysis IV (plant cytosol)	6-phosphofructo-kinase	Dey and Harborne (1997)	$44 \rightarrow 43$
E17	1. galactose degradation II<br>2. galactose degradation III<br>3. sucrose biosynthesis<br>4. UDP-glucose biosynthesis (from glucose 6-phosphate)<br>5. sucrose degradation III<br>6. superpathway of sucrose and starch metabolism I (non-photosynthetic tissue)<br>7. superpathway of sucrose and starch metabolism II (photosynthetic tissue)	UDP-glucose pyro-phosphorylase<br>UDP-D-galactose phosphorylase	Guy et al. (2008)<br>Reiter (2008)	$50 + 129 \rightarrow 82$<br>$76 + 129 \rightarrow 77$
E18_f<br>E18_b	1. galactose degradation III<br>2. sucrose biosynthesis<br>3. UDP-glucose biosynthesis (from sucrose)<br>4. sucrose degradation III	sucrose synthase	Guy et al. (2008)	$66 + 115 \rightarrow 63 + 82$<br>$63 + 82 \rightarrow 66 + 115$
E19	1. galactose degradation III<br>2. UDP-D-xylose biosynthesis	UDP-D-glucose dehydrogenasae	Reiter (2008)	$82 \rightarrow 85$
E20_b<br>E20_f	1. galactose degradation II<br>2. galactose degradation III<br>3. UDP-galactose biosynthesis<br>4. galactose degradation I (Leloir pathway)	UDP-glucose 4-epimerase	Guy et al. (2008)	$1.77 \rightarrow 82$<br>$2.82 \rightarrow 77$<br>$3.77 \rightarrow\leftarrow 82$<br>$4.77 \rightarrow 82$
E21	Non-enzymatic H_2O_2 interaction	Non-enzymatic	Cousins et al. (2008)	$15 \rightarrow 35$
E22	1. galactose degradation II<br>2. galactose degradation III<br>3. galactose degradation I (Leloir pathway)	D-galactokinase	Reiter (2008)	$73 \rightarrow 76$

Supplementary Table 4. Reactions of the complete PN model. For the legend see Table 2.

Transition	Biochemical pathway	Enzyme name	Reference	Reactions
E23	UDP-D-xylose biosynthesis	UDP-D-glucuronate decarboxylase	Reiter (2008)	$85 \rightarrow 88$
E24_f<br>E24_b	galactose degradation I (Leloir pathway)	ADP glucose-1-phosphate adenylyltransferase	Berg et al. (2002)	$76 + 82 \rightarrow 50 + 77$<br>$77 + 50 \rightarrow 82 + 76$
E25	ascorbate biosynthesis I (L-galactose pathway)	L-galactose dehydrogenase	Davey et al. (1999)	$73 \rightarrow 78$
E26_f<br>E26_b	no pathway assigned in AraCyc	UDP-galacturonate pyrophosphatease	Litterer et al. (2006)	$131 \rightarrow 132 + 129$<br>$132 + 129 \rightarrow 131$
E27	stachyose biosynthesis	galactinol synthase	Guy et al. (2008)	$74 + 77 \rightarrow 72 + 115$
E28	stachyose biosynthesis	raffinose synthase	Guy et al. (2008)	$72 + 66 \rightarrow 74 + 70$
E29	stachyose biosynthesis	stachyose synthase	Guy et al. (2008)	$70 + 72 \rightarrow 71 + 74$
E30	trehalose biosynthesis I	trehalose phosphate synthatase	Paul et al. (2008)	$56 + 82 \rightarrow 84 + 115$
E31	trehalose biosynthesis I	trehalose phosphate phosphatase	Paul et al. (2008)	$84 \rightarrow 83$
E32	trehalose biosynthesis I	trehalase	Paul et al. (2008)	$83 \rightarrow 51 + 57$
E33	myo-inositol biosynthesis	myo-inositol 1-phosphate monophosphatase	Guy et al. (2008)	$69 \rightarrow 74$
E34_f<br>E34_b	1D-myo-inositol hexakisphosphate biosynthesis III (Spirodela polyrrhiza)	myo-inositol-1-phosphate synthase	Guy et al. (2008)	$56 \rightarrow 69$<br>$69 \rightarrow 56$
E35	pyrimidine salvage pathway	uracil phospho-ribosyltransferase	Zrenner et al. (2006)	$24 + 121 \rightarrow 80$
E36_f<br>E36_b	No pathway assigned	unknown	Berg et al. (2002)	$51 + 73 \rightarrow 75$<br>$75 \rightarrow 73 + 51$
E37	sucrose degradation III	vacuolar invertase	Tymowska-Lalanne and Kreis (1998)	$66 \rightarrow 51 + 63$
E38	uridine-5-phosphate biosynthesis	carbamoylphos-phatesynthase	Zrenner et al. (2006)	$33 \rightarrow 2 + 116$
E39	No pathway assigned	aldose 6-phosphate reductase	hypothetical reaction Nosarzewski et al. (2012) enzyme name and reaction Negm and Loescher (1981)	$49 \rightarrow 62$
E40	citrulline biosynthesis arginine biosynthesis I arginine biosynthesis II (acetyl cycle)	ornithine transcarbamoylase	Tischner et al. (2007)	$1 + 116 \rightarrow 94$

Supplementary Table 5. Reactions of the complete PN model. For the legend see Table 2.

Transition	Biochemical pathway	Enzyme name	Reference	Reactions
E41	no pathway assigned			
E42	uridine-5-phosphate biosynthesis	aspartate transcarbamoylase	Zrenner et al. (2006)	$23 + 116 \rightarrow 117$
E43	uridine-5-phosphate biosynthesis	dihydroorotase	Zrenner et al. (2006)	$117 \rightarrow 118$
E44	uridine-5-phosphate biosynthesis	dihydroorotate dehydrogenase	Zrenner et al. (2006)	$118 \rightarrow 119$
E45_73<br>E45_66	no pathway assigned	alpha-galactosidase	Gross and Pharr (1982)	$71 \rightarrow 70 + 73$<br>$70 \rightarrow 66 + 73$
E46	starch biosynthesis	ADP-glucose pyrophosphorylase	Kossmann and Lloyd (2000)	$50 \rightarrow 58$
E47	not assigned			
E48	not assigned			
E49	not assigned			
E50	not assigned			
E51	not assigned			
E52	not assigned			
E53	not assigned			
E54_b<br>E54_f	connection of glycolysis and pentosephosphate pathway	transaldolase	Berg et al. (2002)	$32 + 44 \rightarrow 42 + 65$<br>$65 + 42 \rightarrow 44 + 32$
E55	not assigned			
E56	not assigned			
E57	not assigned			
E58	not assigned			
E59	not assigned			
E60	not assigned			
E61_119<br>E61_80	uridine-5-phosphate biosynthesis	UMP synthase (orotate phosphoribosyltransferase)	Zrenner et al. (2006)	$119 + 121 \rightarrow 120$<br>$120 \rightarrow 80$
E62_u<br>E62_c	pyrimidine ribonucleotides interconversion	uridylate/cytidylate kinase	Zrenner et al. (2006)	$80 \rightarrow 115$<br>$123 \rightarrow 128$

Supplementary Table 6. Reactions of the complete PN model. For the legend see Table 2.

Transition	Biochemical pathway	Enzyme name	Reference	Reactions
E63_b<br>E63_f	glycolysis	enolase	Plaxton (1996)	$39 \rightarrow 34$<br>$34 \rightarrow 39$
E64	chorismate biosynthesis	3-deoxy-arabino-heptulonate 7-phosphate (DAHP) synthase	Herrmann (1995)	$32 + 34 \rightarrow 36$
E65	chorismate biosynthesis	3-dehydroquinate synthase	Herrmann (1995)	$36 \rightarrow 37$
E66_95_b<br>E66_95_f<br>E66_101_f<br>E66_101_b	chorismate biosynthesis	1. 3-dehydroquinate dehydratase<br>2. quinate hydrolyase	Herrmann (1995)	1. $37 \rightarrow 95$<br>1. $95 \rightarrow 37$<br>2. $101 \rightarrow 102$<br>2. $102 \rightarrow 101$
E67_101_f<br>E67_101_b<br>E67_95_f<br>E67_95_b	chorismate biosynthesis	1. quinate dehydrogenase<br>2. shikimate dehydrogenase<br>3. shikimate kinase	Herrmann (1995)	1. $37 \rightarrow 101$<br>1. $101 \rightarrow 37$<br>2. $95 \rightarrow 102$<br>2. $102 \rightarrow 95$<br>3. $102 \rightarrow 96$
E68	chorismate biosynthesis	5-enolpyruvylshikimate 3-phosphate (EPSP) synthase	Herrmann (1995)	$34 + 96 \rightarrow 97$
E69	chorismate biosynthesis	chorismate synthase	Herrmann (1995)	$97 \rightarrow 103$
E70	chorismate biosynthesis	chorismate mutase	Strawn et al. (2006)	$103 \rightarrow 105$
E71_f<br>E71_b	phenylalanine biosynthesis II<br>tyrosine biosynthesis II	prephenate amino-transferase	Graindorge et al. (2010)	$2 + 105 \rightarrow 9 + 98$<br>$9 + 98 \rightarrow 2 + 105$
E72_107<br>E72_106	tyrosine<br>biosynthesis II	1. arogenate dehydrogenase<br>2. prephenate dehydro genase	1. Rippert and Matringe (2002)<br>2. hypothetical Rippert and Matringe (2002)	1. $98 \rightarrow 107$<br>2. $105 \rightarrow 106$
E73	phenylalanine biosynthesis II	arogenate dehydratase	Cho et al. (2007)	$98 \rightarrow 109$
E74	tyrosine degradation I	4-hydroxyphenylpyruvate dioxygenase	Garcia et al. (1999)	$106 \rightarrow 99$
E75_f<br>E75_b	tyrosine biosynthesis I	tyrosine amino-transferase	Tao et al. (2008)<br>Holländer-Czytko et al. (2005)	$9 + 107 \rightarrow 2 + 106$<br>$2 + 106 \rightarrow 9 + 107$
E76	Not assigned			
E77	phenylpropanoid biosynthesis	phenylalanine ammonialyase	Humphreys and Chapple (2002)	$109 \rightarrow 110 + 29$
E78	phenylpropanoid biosynthesis	phenylalanine ammonialyase	Humphreys and Chapple (2002)	$109 \rightarrow 110 + 29$

Supplementary Table 7. Reactions of the complete PN model. For the legend see Table 2.

Transition	Biochemical pathway	Enzyme name	Reference	Reactions
E79	phenylpropanoid biosynthesis	cinnamate 4-hydroxylase	Humphreys and Chapple (2002)	$110 \rightarrow 112$
E80	phenylpropanoid biosynthesis	4-(hydroxy)cinnamoyl CoA ligase	Humphreys and Chapple (2002)	$112 + 93 \rightarrow 47$
E81_111<br>E81_104<br><br>E81_90<br>E81_100	phenylpropanoid biosynthesis	1. hydroxycinnamoyl CoA:quinate hydroxy-cinnamoyltransferase<br>2. hydroxycinnamoyl CoA:shikimate hydroxy-cinnamoyltransferase	Humphreys and Chapple (2002)	1. $93 + 111 \rightarrow 101 + 108$ &<br>$47 + 101 \rightarrow 93 + 104$<br><br>2. $90 + 93 \rightarrow 102 + 108$ &<br>$47 + 102 \rightarrow 93 + 100$
E82_90<br>E82_111	phenylpropanoid biosynthesis	p-coumarate 3-hydroxylase	Humphreys and Chapple (2002)	$100 \rightarrow 90$<br>$104 \rightarrow 111$
E83	phenylpropanoid biosynthesis	caffeoyl CoA O-methyltransferase	Humphreys and Chapple (2002)	$108 \rightarrow 7$
E84	phenylpropanoid biosynthesis	cinnamoyl CoA reductase	Humphreys and Chapple (2002)	$7 \rightarrow 17 + 93$
E85	phenylpropanoid biosynthesis	cinnamyl alcohol dehydrogenase / sinapyl alcohol dehydrogenase	Humphreys and Chapple (2002)	$17 \rightarrow 19$
E86_31<br>E86_133	phenylpropanoid biosynthesis	ferulate 5-hydroxylase	Humphreys and Chapple (2002)	$19 \rightarrow 31$ &<br>$17 \rightarrow 133$
E87_48<br>E87_134	phenylpropanoid biosynthesis	caffeic acid/5-hydroxyferulic acid Omethyltransferase	Humphreys and Chapple (2002)	$31 \rightarrow 48$ &<br>$133 \rightarrow 134$
E88_19<br>E88_48	phenylpropanoid biosynthesis		Humphreys and Chapple (2002)	$19 \rightarrow 30a$ &<br>$48 \rightarrow 30b$<br>(two different kinds of lignin)
E89_f<br><br>E89_b	1. glycolysis IV (plant cytosol)<br>2. gluconeogenesis<br>3. glycolysis I (plastidic)<br>4. rubisco shunt	phosphoglyceromutase	Plaxton (1996)	1. $40 \rightarrow 39$<br><br>2. $39 \rightarrow 40$<br>3. $39 \rightarrow 40$<br><br>4. $40 \rightarrow 39$
E90_f<br><br>E90_b	1. glycolysis IV (plant cytosol)<br>2. gluconeogenesis<br>3. Calvin-Benson-Bassham cycle<br>4. glycolysis I (plastidic)	3-PGA kinase	Plaxton (1996)	1. $41 \rightarrow 40$<br><br>2. $40 \rightarrow 41$<br>3. $40 \rightarrow 41$<br><br>4. $40 \rightarrow 41$
E91_b<br><br>E91_f	1. Calvin-Benson-Bassham cycle<br>2. gluconeogenesis	NAD-dependent GAPDH (phosphorylating)	Plaxton (1996)	1. $41 \rightarrow 42$<br><br>2. $41 \rightarrow 42$

Supplementary Table 8. Reactions of the complete PN model. For the legend see Table 2.

Transition	Biochemical pathway	Enzyme name	Reference	Reactions
E92	CO2 fixation into oxaloacetate glutamine biosynthesis III	phosphoenolpyruvate carboxylase	Dey and Harborne (1997)	$34 \rightarrow 12$
E93	gluconeogenesis superpathway of glyoxylate cycle	phosphoenolpyruvate carboxylase	Cornah et al. (2004)	$12 \rightarrow 34$
E94_f<br>E94_b	TCAcycle<br>glyoxylate cycle	malate dehydrogenase	Berg et al. (2002)<br>Cornah et al. (2004)	$8 \rightarrow 12$<br>$12 \rightarrow 8$
E95	sinapate ester biosynthesis	hydroxycinnamaldehyde dehydrogenase	Fraser and Chapple (2011)	$134 \rightarrow 55$
E96_b<br>E96_f	TCAcycle	fumarate hydratase	Berg et al. (2002)<br>Cornah et al. (2004)	$6 \rightarrow 8$<br>$8 \rightarrow 6$
E97_f<br>E97_b	TCAcycle	succinate dehydrogenase	Berg et al. (2002)<br>Cornah et al. (2004)	$4 \rightarrow 6$<br>$6 \rightarrow 4$
E98	TCAcycle<br>glyoxylate cycle	citrate synthase	Berg et al. (2002)<br>Cornah et al. (2004)	$12 + 92 \rightarrow 11 + 93$
E99	TCAcycle<br>glyoxylate cycle	aconitase	Berg et al. (2002)<br>Cornah et al. (2004)	$11 \rightarrow 10$
E100	TCAcycle	isocitrate dehydrogenase	Berg et al. (2002)<br>Cornah et al. (2004)	$10 \rightarrow 9$
E101	TCAcycle	a-ketoglutarate dehydrogenase	Berg et al. (2002)<br>Aubert et al. (2001)	$9 + 93 \rightarrow 5$
E102_f<br>E102_b	TCAcycle	succinate-CoA ligase	Berg et al. (2002)<br>Cornah et al. (2004)	$5 \rightarrow 4 + 93$<br>$4 + 93 \rightarrow 5$
E103	glutamate degra degradation IV 4-aminobutyrate degradation IV	succinic semialdehyde dehydrogenase	Bouché and Fromm (2004)	$79 \rightarrow 4$
E104_f<br>E104_b	glutamate degra-degradation IV 4-aminobutyrate degradation IV	gamma-aminobutyric acid transaminase	Bouché and Fromm (2004)	$3 + 20 \rightarrow 21 + 79$
E105	glutamate degradation IV 4-aminobutyrate degradation IV	glutamate decarboxylase	Bouché and Fromm (2004)	$2 \rightarrow 3$
E106_f<br>E106_b	glutamate degradation I	glutamate dehydrogenase	Aubert et al. (2001)	$9 + 29 \rightarrow 2$<br>$2 \rightarrow 29 + 9$

Supplementary Table 9. Reactions of the complete PN model. For the legend see Table 2.

Transition	Biochemical pathway	Enzyme name	Reference	Reactions
E107	glutamate biosynthesis V ammonia assimilation cycle II glutamine biosynthesis III	glutamate synthase	Forde and Lea (2007)	$9 + 33 \to 2x2$
E108	glutamate biosynthesis V ammonia assimilation cycle II glutamine biosynthesis III	glutamate synthase	Forde and Lea (2007)	$9 + 33 \to 2x2$
E109	ammonia assimilation cycle II glutamine biosynthesis I glutamine biosynthesis III	glutamine synthetase	Forde and Lea (2007)	$2 + 29 \to 33$
E110	glutamine biosynthesis III	pyruvate, orthophosphate dikinase	Matsuoka (1995)	$20 \to 34$
E111	glycolysis IV (plant cytosol) glycolysis I (plastidic) rubisco shunt	pyruvate kinase	Andre et al. (2007)	$34 \to 20$
E112	sucrose degradation to ethanol and lactate (anaerobic) pyruvate fermentation to ethanol II acetaldehyde biosynthesis I	pyruvate decarboxylase	Ismond et al. (2003)	$20 \to 114$
E113_b	1. alanine degradation III<br>1. alanine biosynthesis II<br>2. glycine biosynthesis<br>2. photorespiration	1. alanine aminotransferase<br>2. glyoxylate aminotransferase	Igarashi et al. (2003)	1. $2 + 20 \to 9 + 21$<br>$9 + 21 \to 2 + 20$<br>2. $2 + 13 \to 9 + 18$
E114	ethanol degradation II (cytosol) ethanol degradation IV (peroxisomal)	acetate-CoA ligase	Lin and Oliver (2008)	$38 + 93 \to 92$
E115	oxidative ethanol degradation III oxidative ethanol degradation I	aldehyde dehydrogenase	Wei et al. (2007)	$114 \to 38$
E116	cysteine biosynthesis I	O-acetylserine (thiol) lyase	Wirtz and Hell (2006)	$91 \to 38 + 113$

Supplementary Table 10. Reactions of the complete PN model. For the legend see Table 2.

Transition	Biochemical pathway	Enzyme name	Reference	Reactions
E117	cysteine biosynthesis I	serine acetyltransferase	Wirtz and Hell (2006)	$16 + 92 \rightarrow 91 + 93$
E118	glycine biosynthesis	serine hydroxy-methyltransferase	Berg et al. (2002)	$16 + 29 \rightarrow 2x18$
E119	glycine biosynthesis photorespiration	serine hydroxy-methyltransferase	Goyer et al. (2005)	$2x18 \rightarrow 16 + 29$
E120_u<br>E120_c	pyrimidine salvage pathway	pyrimidine specific 5-nucleotidase	Zrenner et al. (2006)	$80 \rightarrow 126$<br>$123 \rightarrow 127$
E121	glycine biosynthesis photorespiration	glyoxylate aminotransferase	Liepman and Olsen (2001)	$13 + 16 \rightarrow 15 + 18$
E122	photorespiration	hydroxypyruvate reductase	Cousins et al. (2008)	$15 \rightarrow 14$
E123	photorespiration	glycerate kinase	Liepman and Olsen (2003)	$14 \rightarrow 40$
E124	Not assigned			
E125	glyoxylate cycle	isocitrate lyase	Cornah et al. (2004)	$10 \rightarrow 4 + 13$
E126	glycolate and glyoxylate degradation II	glycolate oxidase	Liepman and Olsen (2003)	$35 \rightarrow 13$
E127	uridine-5-phosphate biosynthesis	5-phosphoribosyl-1-pyrophosphate synthase	Zrenner et al. (2006)	$46 \rightarrow 121$
E128	photorespiration	phosphoglycolate phosphatase	Linka and Weber (2005)	$64 \rightarrow 35$
E129	photorespiration ribulose<br><br>Rubisco shunt/ Calvin-Benson-Bassham cycle	1,5-bisphosphate carboxlyase / oxygenase (Rubisco)	Linka and Weber (2005)	$60 \rightarrow 40 + 64$<br><br>$3x60 \rightarrow 6x40$
E130	Calvin-Benson-Bassham cycle rubisco shunt	phosphoribulo-kinase	Berg et al. (2002)	$52 \rightarrow 60$
E131	pyrimidine salvage pathway pyrimidine ribonucleosides degradation II	uridine nucleosidase (+ ribokinase for ribose $\rightarrow$ 46)	Zrenner et al. (2006)	$126 \rightarrow 24 + 46$
E132	uracil degradation (reductive)	dihydrouracil dehydrogenase	Zrenner et al. (2006)	$24 \rightarrow 124$
E133_f<br>E133_b	citrulline biosynthesis<br>arginine biosynthesis I<br>arginine biosynthesis II (acetyl cycle)<br>urea cycle	argininosuccinate lyase	Tischner et al. (2007)	$27 \rightarrow 6 + 28$<br>$6 + 28 \rightarrow 27$

Supplementary Table 11. Reactions of the complete PN model. For the legend see Table 2.

Transition	Biochemical pathway	Enzyme name	Reference	Reactions
E134	citrulline biosynthesis<br>arginine biosynthesis I<br>arginine biosynthesis II (acetyl cycle)<br>urea cycle	argininosuccinate synthetase	Tischner et al. (2007)	$23 + 94 \rightarrow 27$
E135	citrulline biosynthesis<br>arginine biosynthesis I<br>arginine biosynthesis II (acetyl cycle)<br>urea cycle<br>arginine degradation VI (arginase 2 pathway)	arginase	Tischner et al. (2007)	$28 \rightarrow 1 + 25$
E136	urea degradation II	urease	Sirko and Brodzik (2000)	$25 \rightarrow 2x29$
E137	uracil degradation (reductive)	dihydropyrimidinase	Zrenner et al. (2006)	$124 \rightarrow 125$
E138	uracil degradation (reductive)	b-ureidopropionase	Zrenner et al. (2006)	$125 \rightarrow 29$<br>beta-alanine not modeled, reaction only recycles bound NH3, which would be set free in the reaction to beta-alanine
E139_u_dtom<br>E139_c_dtom<br>E139_u_ttod<br>E139_c_ttod	pyrimidine ribonucleotides interconversion	1. nucleoside di-phosphate phosphatase<br>2. nucleoside tri-phosphate phosphatase	Zrenner et al. (2006)	1. $115 \rightarrow 80$<br>$128 \rightarrow 123$<br>2. $129 \rightarrow 115$<br>$122 \rightarrow 128$
E140	asparagine biosynthesis I	asparagine synthetase	Lam et al. (1994)	$23 + 33 \rightarrow 2 + 26$
E141	asparagine degradation I	asparaginase	Bruneau et al. (2006)	$26 \rightarrow 23 + 29$
E142	Not assigned			
E143_f<br>E143_b	No pathway assigned	aldonolactonase	Ishikawa and Shigeoka (2008)	$130 \rightarrow 78$<br>$78 \rightarrow 130$
E144_u<br>E144_c	pyrimidine ribonucleotides interconversion	nucleoside diphos-phate kinase	Zrenner et al. (2006)	$115 \rightarrow 129$<br>$128 \rightarrow 122$
E145_u<br>E145_c	pyrimidine ribonucleotides interconversion	apyrase	Zrenner et al. (2006)	$129 \rightarrow 80$<br>$122 \rightarrow 123$
E146	pyrimidine ribonucleotides interconversion	CTP synthase	Zrenner et al. (2006)	$33 + 129 \rightarrow 2 + 122$

Supplementary Table 12. Reactions of the complete PN model. For the legend see Table 2.

Transition	Biochemical pathway	Enzyme name	Reference	Reactions
E147	pyrimidine ribonucleotides interconversion	cytidine deaminase	Zrenner et al. (2006)	$127 \rightarrow 29 + 126$
E148_b<br><br><br>E148_f	1. pentose phosphate pathway (non-oxidative branch)<br>2. Calvin-Benson-Bassham cycle<br>3. Rubisco shunt	ribulose-phosphate 3-epimerase	Berg et al. (2002)<br>Dey and Harborne (1997)	1. $52 \rightarrow 45$<br><br><br>2. $45 \rightarrow 52$
E149	no pathway assigned	sorbitol dehydro-genase	Nosarzewski et al. (2012)	$62 \rightarrow 63$
E150	no pathway assigned	UDP-glucuronate 4-epimerase	Ishikawa and Shigeoka (2008)	$85 \rightarrow 131$
E151	no pathway assigned	UDP-galacturonate pyrophosphatase	Ishikawa and Shigeoka (2008)	$132 \rightarrow 87$
E152	no pathway assigned	D-galacturonate reductase	Ishikawa and Shigeoka (2008)	$87 \rightarrow 130$
E153	phenylpropanoid biosynthesis	hydroxycinnamalde-hyde dehydrogenase	Fraser and Chapple (2011)	$134 \rightarrow 48$
E154	glycolysis	lipoate acetyl-transferase	Buchanan et al. (2000)	$20 + 93 \rightarrow 92$
E155	glyoxylate cycle	malate synthase	Buchanan et al. (2000)	$13 + 92 \rightarrow 8 + 93$
IN_18	glycine		Jander et al. (2004)<br>Joshi et al. (December 2006)	$\rightarrow 18$
IN_29	ammonia		von Wirén et al. (2000)<br>Morgan and Jackson (1988)	$\rightarrow 29$
IN_63	D-fructose		Sauer et al. (1990)	$\rightarrow 63$
IN_73	D-galactose		Sauer et al. (1990)	$\rightarrow 73$
IN_92	acetyl-CoA		Fulda et al. (2002)	$\rightarrow 92$
IN_93	CoA		Kupke et al. (2001)	$\rightarrow 93$
IN_94	citrulline		Ludwig (1993)	$\rightarrow 94$
OUT_29	ammonia		Morgan and Jackson (1988)	$29 \rightarrow$
OUT_30a	lignin		Buchanan et al. (2000)	$30a \rightarrow$
OUT_30b	lignin		Buchanan et al. (2000)	$30b \rightarrow$
OUT_50	Alpha-D-glucose 1-phosphate		Wolucka and Montagu (2007)	$50 \rightarrow$

Supplementary Table 13. Reactions of the complete PN model. For the legend see Table 2.

Transition	Biochemical pathway	Enzyme name	Reference	Reactions
OUT_51	Beta-D-glucose		AraCyc (2012)	$51 \rightarrow$
OUT_62	sorbitol		Noiraud et al. (2001)	$62 \rightarrow$
OUT_66	sucrose		Sauer and Stolz (1994)	$66 \rightarrow$
OUT_71	stachyose		AraCyc (2012)	$71 \rightarrow$
OUT_74	D-myo-inositol		Reiter (2008)	$74 \rightarrow$
OUT_78	L-galactono-1,4-lactone		Davey et al. (1999)	$78 \rightarrow$
OUT_82	UDP-glucose		Dean and Delaney (2008)	$82 \rightarrow$
OUT_88	UDP-xylose		Buchanan et al. (2000)	$88 \rightarrow$
OUT_92	acetyl-CoA		external metabolite	$92 \rightarrow$
OUT_93	CoA		external metabolite	$93 \rightarrow$
OUT_94	citrulline		external metabolite	$94 \rightarrow$
OUT_99	homogentisate		Collakova and DellaPenna (2001)	$99 \rightarrow$
OUT_103	chorismate		Strawn et al. (2006)	$103 \rightarrow$
OUT_106	4-hydroxy-phenylpyrovate		AraCyc (2012)	$106 \rightarrow$
OUT_113	cysteine		Buchanan et al. (2000)	$113 \rightarrow$
OUT_115	uridine diphosphate (UDP)		Buchanan et al. (2000)	$115 \rightarrow$
OUT_122	CTP		Zrenner et al. (2006)	$122 \rightarrow (RNAsynthesis)$
OUT_129	UTP		Zrenner et al. (2006)	$129 \rightarrow (RNAsynthesis)$
R1	starch degradation	phosphoglucan, water dikinase	Guy et al. (2008)	$59 \rightarrow 61$
R2	starch degradation	glucan, water dikinase	Fettke et al. (2009)	$59 \rightarrow 2x57$
R3	starch synthesis	starch branching enzyme	Kossmann and Lloyd (2000)	$81 + 86 \rightarrow 59$
R4_b<br>R4_f	starch degradation	disproportionating isozyme 2	Fettke et al. (2009)	$61 \rightarrow 57 + 89$<br>$57 + 89 \rightarrow 61$
R5_1<br>R5_2	starch synthesis	1. R5 1: granule bound starch synthase<br>2. R5 2: starch synthase	Kossmann and Lloyd (2000)	1. $58 \rightarrow 86$<br>2. $58 \rightarrow 81$
R6_b<br>R6_f	starch degradation	phosphorylase isozyme	Fettke et al. (2009)	$89 \rightarrow 50$<br>$50 \rightarrow 89$

Supplementary Table 14. Output reactions of the PN ordered by the subnetwork and increasing place numbers.

Place number	Metabolite	Subnetwork
29	ammonia	citrate
92	acetyl-CoA	citrate
93	CoA	citrate
94	citrulline	citrate
113	cysteine	citrate
30a	lignin	phenyl
30b	lignin	phenyl
99	homogentisate	phenyl
103	chorismate	phenyl
106	4-hydroxy-phenylpyrovate	phenyl
115	uridine diphosphate (UDP)	UTP
122	cytosine triphosphate (CTP)	UTP
129	uridine triphosphate (UTP)	UTP
50	α-D-glucose 1-phosphate	sucrose
51	β-D-glucose	sucrose
62	sorbitol	sucrose
66	sucrose	sucrose
71	stachyose	sucrose
74	D-myo-inositol	sucrose
78	L-galactono-1,4-lactone	sucrose
82	UDP-glucose	sucrose
88	UDP-xylose	sucrose

REFERENCES

Buchanan BB, Gruissem W, Jones RL. *Biochemistry & molecular biology of plants* (American Society of Plant Physiologists, Rockville, Md.) (2000).

Wilkie SE, Warren MJ. Recombinant Expression, Purification, and Characterization of Three Isoenzymes of Aspartate Aminotransferase from Arabidopsis thaliana. *Protein Expr Purif* **12** (1998) 381 – 389. doi:10.1006/prep.1997.0845.

Graindorge M, Giustini C, Jacomin AC, Kraut A, Curien G, Matringe M. Identification of a plant gene encoding glutamate/aspartate-prephenate aminotransferase: the last homeless enzyme of aromatic amino acids biosynthesis. *FEBS Lett* **584** (2010) 4357 – 4360. doi:10.1016/j.febslet.2010.09.037.

Berg JM, Tymoczko JL, Stryer L. *Biochemistry* (W. H. Freeman, New York), 5th edn. (2002).

Dey P, Harborne J, editors. *Plant Biochem* (Academic Press, San Diego, USA) (1997).

Lefebvre S, Lawson T, Zakhleniuk O, Lloyd J, Raines C, Fryer M. Increased Sedoheptulose-1,7-Bisphosphatase Activity in Transgenic Tobacco Plants Stimulates Photosynthesis and Growth from an Early Stage in Development. *Plant Physiol* **138** (2005) 451–460.

Nelson DL, Cox MM. *Lehninger principles of biochemistry* (W. H. Freeman, New York), 3rd edn. (2000).

Reiter W. Biochemical genetics of nucleotide sugar interconversion reactions. *Curr Opin Plant Biol* **11** (2008) 236–243.

Lu Y, Sharkey T. The importance of maltose in transitory starch breakdown. *Plant, Cell & Env* **29** (2006) 353–366.

Guy C, Kaplan F, Kopka J, Selbig J, Hincha DK. Metabolomics of temperature stress. *Physiologia Plantarum* **132** (2008) 220–235. doi:10.1111/j.1399-3054.2007.00999.x.

Cousins A, Pracharoenwattana I, Zhou W, Smith S, Badger M. Peroxisomal Malate Dehydrogenase Is Not Essential for Photorespiration in Arabidopsis But Its Absence Causes an Increase in the Stoichiometry of Photorespiratory $CO_2$2 Release. *Plant Physiol* **148** (2008) 786–95.

Davey M, Gilot C, Persiau G, Ostergaard J, Han Y, Bauw G, et al. Ascorbate biosynthesis in Arabidopsis cell suspension culture. *Plant Physiol* **121** (1999) 535–543.

Litterer L, Schnurr J, Plaisance K, Storey K, Gronwald J, Somers D. Characterization and expression of Arabidopsis UDP-sugar pyrophosphorylase. *Plant Physiol Biochem* **44** (2006) 171 – 180. doi:10.1016/j.plaphy.2006.04.004.

Paul MJ, Primavesi LF, Jhurreea D, Zhang Y. Trehalose Metabolism and Signaling. *Annu Rev Plant Biol* **59** (2008) 417–441. doi:10.1146/annurev.arplant.59.032607.092945.

Zrenner R, Stitt M, Sonnewald U, Boldt R. Pyrimidine and purine biosynthesis and degradation in plants. *Annu Rev Plant Biol* **57** (2006) 805–836. doi:10.1146/annurev.arplant.57.032905.105421.

Tymowska-Lalanne Z, Kreis M. Expression of the Arabidopsis thaliana invertase gene family. *Planta* **207** (1998) 259–265. 10.1007/s004250050481.

Nosarzewski M, Downie AB, Wu B, Archbold DD. The role of sorbitol dehydrogenase in Arabidopsis thaliana. *Funct Plant Biol* **39** (2012) 462–470.

Negm FB, Loescher WH. Characterization and Partial Purification of Aldose-6-phosphate Reductase (Alditol-6-Phosphate:NADP 1-Oxidoreductase) from Apple Leaves. *Plant Physiol* **67** (1981) 139–142.

Tischner R, Galli M, Heimer YM, Bielefeld S, Okamoto M, Mack A, et al. Interference with the citrulline-based nitric oxide synthase assay by argininosuccinate lyase activity in Arabidopsis extracts. *FEBS J* **274** (2007) 4238–4245. doi:10.1111/j.1742-4658.2007.05950.x.

Gross KC, Pharr DM. A Potential Pathway for Galactose Metabolism in Cucumis sativus L., A Stachyose Transporting Species. *Plant Physiol* **69** (1982) 117–121. doi:10.1104/pp.69.1.117.

Kossmann J, Lloyd J. Understanding and Influencing Starch Biochemistry. *Crit Rev Biochem Mol Biol* **35** (2000) 141–196.

Plaxton WC. THE ORGANIZATION AND REGULATION OF PLANT GLYCOLYSIS. *Annu Rev Plant Physiol Plant Mol Biol* **47** (1996) 185–214. doi:10.1146/annurev.arplant.47.1.185.

Herrmann KM. The Shikimate Pathway: Early Steps in the Biosynthesis of Aromatic Compounds. *Plant Cell* **7** (1995) 907–919. doi:10.1105/tpc.7.7.907.

Strawn MA, Marr SK, Inoue K, Inada N, Zubieta C, Wildermuth MC. Arabidopsis Isochorismate Synthase Functional in Pathogen-induced Salicylate Biosynthesis Exhibits Properties Consistent with a Role in Diverse Stress Responses. *J Biol Chem* **282** (2006) 5919–5933. doi:10.1074/jbc.M605193200.

Rippert P, Matringe M. Molecular and biochemical characterization of an Arabidopsis thaliana arogenate dehydrogenase with two highly similar and active protein domains. *Plant Mol Biol* **48** (2002) 361–368. doi:10.1023/A:1014018926676.

Cho MH, Corea ORA, Yang H, Bedgar DL, Laskar DD, Anterola AM, et al. Phenylalanine biosynthesis in Arabidopsis thaliana. Identification and characterization of arogenate dehydratases. *J Biol Chem* **282** (2007) 30827–30835. doi:10.1074/jbc.M702662200.

Garcia I, Rodgers M, Pepin R, Hsieh TF, Matringe M. Characterization and Subcellular Compartmentation of Recombinant 4-Hydroxyphenylpyruvate Dioxygenase from Arabidopsis in Transgenic Tobacco. *Plant Physiol* **119** (1999) 1507–1516. doi:10.1104/pp.119.4.1507.

Tao Y, Ferrer JL, Ljung K, Pojer F, Hong F, Long JA, et al. Rapid Synthesis of Auxin via a New Tryptophan-Dependent Pathway Is Required for Shade Avoidance in Plants. *Cell* **133** (2008) 164 – 176. doi:10.1016/j.cell.2008.01.049.

Holländer-Czytko H, Grabowski J, Sandorf I, Weckermann K, Weiler EW. Tocopherol content and activities of tyrosine aminotransferase and cystine lyase in Arabidopsis under stress conditions. *J Plant Physiol* **162** (2005) 767 – 770. doi:10.1016/j.jplph.2005.04.019.

Humphreys JM, Chapple C. Rewriting the lignin roadmap. *Curr OpinPlant Biol* **5** (2002) 224 – 229. doi:10.1016/S1369-5266(02)00257-1.

Cornah JE, Germain V, Ward JL, Beale MH, Smith SM. Lipid Utilization, Gluconeogenesis, and Seedling Growth in Arabidopsis Mutants Lacking the Glyoxylate Cycle Enzyme Malate Synthase. *J Biol Chem* **279** (2004) 42916–42923.

Fraser CM, Chapple C. The Phenylpropanoid Pathway in Arabidopsis. *The Arabidopsis Book* (American Society of Plant Biologists), vol. 9 (2011). Epub ahead of print: Dec 6, 2011.

Aubert S, Bligny R, Douce R, Gout E, Ratcliffe R, Roberts J. Contribution of glutamate dehydrogenase to mitochondrial glutamate metabolism studied by (13)C and (31)P nuclear magnetic resonance. *J Exp Bot* **52** (2001) 37–45.

Bouché N, Fromm H. GABA in plants: just a metabolite? *Trends Plant Sci* **9** (2004) 110 – 115. doi:10.1016/j.tplants.2004.01.006.

Forde BG, Lea PJ. Glutamate in plants: metabolism, regulation, and signalling. *J Exp Bot* **58** (2007) 2339–2358.

Matsuoka M. The Gene for Pyruvate,Orthophosphate Dikinase in C4 Plants: Structure, Regulation and Evolution. *Plant Cell Physiol* **36** (1995) 937–943.

Andre C, Froehlich JE, Moll MR, Benning C. A Heteromeric Plastidic Pyruvate Kinase Complex Involved in Seed Oil Biosynthesis in Arabidopsis. *Plant Cell* **19** (2007) 2006–2022. doi:10.1105/tpc.106.048629.

Ismond KP, Dolferus R, De Pauw M, Dennis ES, Good AG. Enhanced Low Oxygen Survival in Arabidopsis through Increased Metabolic Flux in the Fermentative Pathway. *Plant Physiol* **132** (2003) 1292–1302. doi:10.1104/pp.103.022244.

Igarashi D, Miwa T, Seki M, Kobayashi M, Kato T, Tabata S, et al. Identification of photorespiratory glutamate: glyoxylate aminotransferase (GGAT) gene in Arabidopsis. *Plant J* **33** (2003) 975–987. doi:10.1046/j.1365-313X.2003.01688.x.

Lin M, Oliver DJ. The Role of Acetyl-Coenzyme A Synthetase in Arabidopsis. *Plant Physiol* **147** (2008) 1822–1829. doi:10.1104/pp.108.121269.

Wei Y, Lin M, Oliver D, Schnable P. The roles of aldehyde dehydrogenases (ALDHs) in the PDH bypass of Arabidopsis. *BMC Biochem* **10** (2007). doi:10.1186/1471-2091-10-7.

Wirtz M, Hell R. Functional analysis of the cysteine synthase protein complex from plants: structural, biochemical and regulatory properties. *J Plant Physiol* **163** (2006) 273 – 286. doi:10.1016/j.jplph.2005.11.013.

Goyer A, Collakova E, de la Garza RD, Quinlivan EP, Williamson J, Gregory JF III, et al. 5-Formyltetrahydrofolate Is an Inhibitory but Well Tolerated Metabolite in Arabidopsis Leaves. *J Biol Chem* **280** (2005) 26137–26142. doi:10.1074/jbc.M503106200.

Liepman AH, Olsen LJ. Peroxisomal alanine: glyoxylate aminotransferase (AGT1) is a photorespiratory enzyme with multiple substrates in Arabidopsis thaliana. *Plant J* **25** (2001) 487–498. doi:10.1046/j.1365-313x.2001.00961.x.

Liepman AH, Olsen LJ. Alanine Aminotransferase Homologs Catalyze the Glutamate: Glyoxylate Aminotransferase Reaction in Peroxisomes of Arabidopsis. *Plant Physiol* **131** (2003) 215–227. doi:10.1104/pp.011460.

Linka M, Weber AP. Shuffling ammonia between mitochondria and plastids during photorespiration. *Trends Plant Sci* **10** (2005) 461 – 465. doi:10.1016/j.tplants.2005.08.002.

Sirko A, Brodzik R. Plant ureases: roles and regulation. *Acta Biochim Polonica* **47** (2000) 1189–9115.

Lam HM, Peng S, Coruzzi GM. Metabolic Regulation of the Gene Encoding Glutamine-Dependent Asparagine Synthetase in Arabidopsis thaliana. *Plant Physiol* **106** (1994) 1347–1357. doi:10.1104/pp.106.4.1347.

Bruneau L, Chapman R, Marsolais F. Co-occurrence of both L-asparaginase subtypes in Arabidopsis: At3g16150 encodes a K^+-dependent L-asparaginase. *Planta* **224** (2006) 668–679. doi:10.1007/s00425-006-0245-9.

Ishikawa T, Shigeoka S. Recent advances in ascorbate biosynthesis and the physiological significance of ascorbate peroxidase in photosynthesizing organisms. *Biosci Biotechnol Biochem* **72** (2008) 1143–1154.

Jander G, Norris SR, Joshi V, Fraga M, Rugg A, Yu S, et al. Application of a high-throughput HPLC-MS/MS assay to Arabidopsis mutant screening; evidence that threonine aldolase plays a role in seed nutritional quality. *Plant J* **39** (2004) 465–475. doi:10.1111/j.1365-313X.2004.02140.x.

Joshi V, Laubengayer KM, Schauer N, Fernie AR, Jander G. Two Arabidopsis Threonine Aldolases Are Nonredundant and Compete with Threonine Deaminase for a Common Substrate Pool. *Plant Cell* **18** (December 2006) 3564–3575. doi:10.1105/tpc.106.044958.

von Wirén N, Gazzarrini S, Gojont A, Frommer WB. The molecular physiology of ammonium uptake and retrieval. *Curr Opin Plant Biol* **3** (2000) 254 – 261. doi:http://dx.doi.org/10.1016/S1369-5266(00)80074-6.

Morgan MA, Jackson WA. Inward and Outward Movement of Ammonium in Root Systems: Transient Responses during Recovery from Nitrogen Deprivation in Presence of Ammonium. *J Exp Bot* **39** (1988) 179–191. doi:10.1093/jxb/39.2.179.

Sauer N, Friedländer K, Gräml-Wicke U. Primary structure, genomic organization and heterologous expression of a glucose transporter from Arabidopsis thaliana. *EMBO J* **9** (1990) 3045.

Fulda M, Shockey J, Werber M, Wolter FP, Heinz E. Two long-chain acyl-CoA synthetases from Arabidopsis thaliana involved in peroxisomal fatty acid β-oxidation. *Plant J* **32** (2002) 93–103. doi:10.1046/j.1365-313X.2002.01405.x.

Kupke T, Hernández-Acosta P, Steinbacher S, Culiáñez-Macià FA. Arabidopsis thaliana Flavoprotein AtHAL3a Catalyzes the Decarboxylation of 4'-Phosphopantothenoylcysteine to 4'-Phosphopantetheine, a Key Step in Coenzyme A Biosynthesis. *J Biol Chem* **276** (2001) 19190–19196. doi:10.1074/jbc.M100776200.

Ludwig RA. Arabidopsis Chloroplasts Dissimilate L-Arginine and L-Citrulline for Use as N Source. *Plant Physiol* **101** (1993) 429–434. doi:10.1104/pp.101.2.429.

Wolucka BA, Montagu MV. The {VTC2} cycle and the de novo biosynthesis pathways for vitamin C in plants: An opinion. *Phytochemistry* **68** (2007) 260–2613. doi:http://dx.doi.org/10.1016/j.phytochem.2007.08.034.

AraCyc (2012). Www.arabidopsis.org/biocyc/.

Noiraud N, Maurousset L, Lemoine R. Transport of polyols in higher plants. *Plant Physiol Biochem* **39** (2001) 717 – 728. doi:http://dx.doi.org/10.1016/S0981-9428(01)01292-X.

Sauer N, Stolz J. SUC1 and SUC2: two sucrose transporters from Arabidopsis thaliana; expression and characterization in baker's yeast and identification of the histidine-tagged protein. *Plant J* **6** (1994) 67–77. doi:10.1046/j.1365-313X.1994.6010067.x.

Dean JV, Delaney SP. Metabolism of salicylic acid in wild-type, ugt74f1 and ugt74f2 glucosyltransferase mutants of Arabidopsis thaliana. *Physiol Plant* **132** (2008) 417–425. doi:10.1111/j.1399-3054.2007.01041.x.

Collakova E, DellaPenna D. Isolation and Functional Analysis of Homogentisate Phytyltransferase from Synechocystis sp. PCC 6803 and Arabidopsis. *Plant Physiol* **127** (2001) 1113–1124. doi:10.1104/pp.010421.

Fettke J, Hejazi M, Smirnova J, Höchel E, Stage M, Steup M. Eukaryotic starch degradation: integration of plastidial and cytosolic pathways. *J Exp Bot* **60** (2009) 2907–2922. doi:10.1093/jxb/erp054.
