# Supplementary material for: Modeling the Metabolism of Arabidopsis thaliana: Application of Network Decomposition and Network Reduction in the Context of Petri Nets
Source: Front Genet. 2017 Jun 30;8:85. doi: 10.3389/fgene.2017.00085 (PMC5491931; doi:10.3389/fgene.2017.00085)
Supplement: Supplementary file 2 [file Table2.PDF]

Supplementary Material: Article TitleA verified Petri model for the metabolism of *Arabidopsis thaliana*: application of network decomposition and network reduction

Ina Koch*[1], Joachim Nöthen[1] and Enrico Schleiff, [1] Shared first authorship
*Correspondence:
Ina Koch, Enrico Schleiff
ina.koch@bioinformatik.uni-frankfurt.de, schleiff@bio.uni-frankfurt.de

First Author 2: joachim.noethen@gmx.de

1 SUPPLEMENTARY TABLES

Supplementary Table 1. Table of reductions. *RT* = reduction type (*CTP* = Common Transition Pair, *ITP* = Invariant Transition Pair, *Parallel* = reduction of parallel transitions, **?**, reduced node = the name of the node after the reduction. The columns t_i/p_i, $pc/t_i, t_j$, and t_j/p_j show t_i, pc, and t_j, if the reduction type is *CTP*, and p_i, t_i and t_j, and p_j, if the reduction type is *ITP* or *Parallel*. The column reduced node holds a transition, if the reduction type is *CTP*, and a place, if the reduction type is *ITP*.

RT	t_i/p_i	$pc/t_i, t_j$	t_j/p_j	Reduced vertex
CTP	E151	→ 87	→ E152	ctp(E151 + E152)
CTP	E23	→ 88	→ E41	ctp(E23 + E41)
CTP	E30	→ 84	→ E31	ctp(E30 + E31)
CTP	ctp(E30+E31)	→ 83	→ E32	ctp(ctp(E30 + E31) + E32)
CTP	R5_2	→ 81	→ R3	ctp(R5_2 + R3)
CTP	R5_1	→ 86	→ ctp(R5_2 + R3)	ctp(R5_1 + ctp(R5_2 + R3))
CTP	E5	→ 53	→ E4	ctp(E5 + E4)
CTP	E129_2	→ 64	→ E128	ctp(E129_2 + E128)
CTP	E122	→ 14	→ E123	ctp(E122 + E123)
CTP	E42	→ 117	→ E43	ctp(E42 + E43)
CTP	E61_119	→ 120	→ E61_80	ctp(E61_119 + E61_80)
CTP	E44	→ 119	→ ctp(E61_119 + E61_80)	ctp(E44 + ctp(E61_119 + E61_80))
CTP	ctp(E42 + E43)	→ 118	→ ctp(E44 + ctp(E61_119 + E61_80))	ctp(ctp(E42 + E43) + ctp(E44 + ctp(E61_119 + E61_80)))
CTP	E132	→ 124	→ E137	ctp(E132 + E137)
CTP	ctp(E132 + E137)	→ 125	→ E138	ctp(ctp(E132 + E137) + E138)
CTP	E98	→ 11	→ E99	ctp(E98 + E99)
CTP	E140	→ 26	→ E141	ctp(E140 + E141)
CTP	E135	→ 1	→ E40	ctp(E135 + E40)
CTP	E117	→ 91	→ E116	ctp(E117 + E116)
CTP	E112	→ 114	→ E115	ctp(E112 + E115)
CTP	ctp(E117 + E116)	→ 113	→ OUT_113	ctp(ctp(E117 + E116)+OUT_113)
CTP	E64	→ 36	→ E65	ctp(E64 + E65)
CTP	ctp(E135 + E40)	→ 25	→ E136	ctp(ctp(E135 + E40) + E136)
CTP	E69	→ 103	→ E70	ctp(E69 + E70)

Supplementary Table 2. Table of reductions. For the legend see Table 1.

RT	t_i/p_i	$pc/t_i, t_j$	t_j/p_j	Reduced vertex
CTP	E67	→ 96	→ E68	ctp(E67 + E68)
CTP	ctp(E67 + E68)	→ 97	→ ctp(E69 + E70)	ctp(ctp(E67 + E68) + ctp(E69 + E70))
CTP	E83	→ 7	→ E84	ctp(E83 + E84)
CTP	E82_90	→ 90	→ E81_90	ctp(E82_90 + E81_90)
CTP	E82_111	→ 111	→ E81_111	ctp(E82_111 + E81_111)
CTP	E81_100	→ 100	→ ctp(E82_90 + E81_90)	ctp(E81_100 + ctp(E82_90 + E81_90))
CTP	E81 104	→ 104	→ ctp(E82_111 + E81_111)	ctp(E81_104 + ctp(E82_111 + E81_111))
CTP	E79	→ 112	→ E80	ctp(E79+E80)
CTP	E36_f	→ 75	→ E36_b	ctp(E36_f + E36_b)
CTP	E95	→ 55	→ OUT_55_ sinapate−esther−synthesis	ctp(E95 + OUT_55_sinapate−esther−synthesis)
CTP	E86_31	→ 31	→ E87_48	ctp(E86_31 + E87_48)
CTP	E88_19	→ 30a	→ OUT_30a	ctp(E88_19 + OUT_30a)
CTP	ctp(E83 + E84)	→ 17	→ E85	ctp(ctp(E83+E84) + E85)
CTP	E86_133	→ 133	→ E87_134	ctp(E86_133 + E87_134)
CTP	E88_48	→ 30b	→ OUT_30b	ctp(E88_48 + OUT_30b)
CTP	E74	→ 99	→ OUT_99	ctp(E74 + OUT_99)
ITP	130<br>130	→ E143_f<br>← E143_b	→ 78<br>← 78	itp(130 + 78)<br>itp(130 + 78)
ITP	82<br>82	→ E20_f<br>← E20_b	→ 77<br>← 77	itp(82 + 77)<br>itp(82 + 77)
ITP	76<br>76	→ E24_f<br>← E24_b	→ 50<br>← 50	itp(76 + 50)<br>itp(76 + 50)
ITP<br><br>ITP	69<br>69<br>49<br>49	→ E34_b<br>← E34_f<br>→ spo_b<br>← spo_f	→ 56<br>← 56<br>→ itp(69 + 56)<br>← itp(69 + 56)	itp(69 + 56)<br>itp(69 + 56)<br>itp(49 + itp(69 + 56))<br>itp(49 + itp(69 + 56))
ITP	itp(49 + itp(69 + 56))<br>itp(49 + itp(69 + 56))	→ E8_f<br>← E8_b	→ 44<br>← 44	itp(itp(49 + itp(69 + 56)) + 44)<br>itp(itp(49 + itp(69 + 56)) + 44)

Supplementary Table 3. Table of reductions. For the legend see Table 1.

RT	t_i/p_i	$pc/t_i, t_j$	t_j/p_j	Reduced vertex
ITP	itp(itp(49 + itp(69 + 56)) + 44)<br>itp(itp(49 + itp(69 + 56)) + 44)	→ E9_f<br>← E9_b	→ itp(76 + 50)<br>← itp(76 + 50)	itp(itp(itp(49 + itp(69 + 56)) + 44) + itp(76 + 50))<br>itp(itp(itp(49 + itp(69 + 56)) + 44) + itp(76 + 50))
ITP	itp(itp(itp(49 + itp(69 + 56)) + 44) + itp(76 + 50))<br>itp(itp(itp(49 + itp(69 + 56)) + 44) + itp(76 + 50))	→ R6_f<br>← R6_b	→ 89<br>← 89	itp(itp(itp(itp(49 + itp(69 + 56)) + 44) + itp(76 + 50)) + 89)<br>itp(itp(itp(itp(49 + itp(69 + 56)) + 44) + itp(76 + 50)) + 89)
ITP	itp(itp(itp(itp(49 + itp(69 + 56)) + 44) + itp(76 + 50)) + 89)<br>itp(itp(itp(itp(49 + itp(69 + 56)) + 44) + itp(76 + 50)) + 89)	→ E16<br>← E15	→ 43<br>← 43	itp(itp(itp(itp(itp(49 + itp(69 + 56)) + 44) + itp(76 + 50)) + 89) + 43)<br>itp(itp(itp(itp(itp(49 + itp(69 + 56)) + 44) + itp(76 + 50))+ 89) + 43)
ITP	68<br>68	→ E13_f<br>← E13_b	→ 42<br>← 42	itp(68 + 42)<br>itp(68 + 42)
ITP	itp(68 + 42)<br>itp(68 + 42)	→ E91_f<br>← E91_b	→ 41<br>← 41	itp(itp(68 + 42) + 41)<br>itp(itp(68 + 42) + 41)
ITP	itp(itp(68 + 42) + 41)<br>itp(itp(68 + 42) + 41)	→ E90_f<br>← E90_b	→ 40<br>← 40	itp(itp(itp(68 + 42) + 41) + 40)<br>itp(itp(itp(68 + 42) + 41) + 40)
ITP	45<br>45	→ E148_f<br>← E148_b	→ 52<br>← 52	itp(45 + 52)<br>itp(45 + 52)
ITP	itp(itp(itp(itp(itp(49 + itp(69 + 56)) + 44) + itp(76 + 50)) + 89) + 43)<br>itp(itp(itp(itp(itp(49 + itp(69 + 56)) + 44) + itp(76 + 50)) + 89) + 43)	→ E6_f<br>← E6_b	→ 54<br>← 54	itp(itp(itp(itp(itp(itp(49 + itp(69 + 56)) + 44) + itp(76 + 50)) + 89) + 43) + 54)<br>itp(itp(itp(itp(itp(itp(49 + itp(69 + 56)) + 44) + itp(76 + 50)) + 89) + 43) + 54)
ITP	itp(45 + 52)<br>itp(45 + 52)	→ E3_b<br>← E3_f	→ 46<br>← 46	itp(itp(45 + 52) + 46)<br>itp(itp(45 + 52) + 46)
ITP	itp(itp(itp(68 + 42) + 41) + 40)<br>itp(itp(itp(68 + 42) + 41) + 40)	→ E89_f<br>← E89_b	→ 39<br>← 39	itp(itp(itp(itp(68 + 42) + 41) + 40) + 39)<br>itp(itp(itp(itp(68 + 42) + 41) + 40) + 39)
ITP	129<br>129	→ E139_u_ttod<br>← E144_u	→ 115<br>← 115	itp(129 + 115)<br>itp(129 + 115)
ITP	itp(129 + 115)<br>itp(129 + 115)	→ E139_u_dtom<br>← E62_u	→ 80<br>← 80	itp(itp(129 + 115) + 80)<br>itp(itp(129 + 115) + 80)
ITP	126<br>126	→ E35_u<br>← E120_u	→ itp(itp(129 + 115) + 80)<br>← itp(itp(129 + 115) + 80)	itp(126 + itp(itp(129 + 115) + 80))<br>itp(126 + itp(itp(129 + 115) + 80))
ITP	128<br>128	→ E139_c_dtom<br>← E62_c	→ 123<br>← 123	itp(128 + 123)<br>itp(128 + 123)
ITP	122<br>122	→ E139_c_ttod<br>← E144_c	→ itp(128 + 123)<br>← itp(128 + 123)	itp(122 + itp(128 + 123))<br>itp(122 + itp(128 + 123))
ITP	itp(122 + itp(128 + 123))<br>itp(122 + itp(128 + 123))	→ E120_c<br>← E35_c	→ 127<br>← 127	itp(itp(122 + itp(128 + 123)) + 127)<br>itp(itp(122 + itp(128 + 123)) + 127)

Supplementary Table 4. Table of reductions. For the legend see Table 1.

RT	t_i/p_i	$pc/t_i, t_j$	t_j/p_j	Reduced vertex
ITP	12	→ E94_b	→ 8	itp(12 + 8)
	12	← E94_f	← 8	itp(12 + 8)
ITP	itp(12 + 8)	→ E93	→ 34	itp(itp(12 + 8) + 34)
	itp(12 + 8)	← E92	← 34	itp(itp(12 + 8) + 34)
ITP	itp(itp(12 + 8) + 34)	→ E111	→ 20	itp(itp(itp(12 + 8) + 34) + 20)
	itp(itp(12 + 8) + 34)	← E110	← 20	itp(itp(itp(12 + 8) + 34) + 20)
ITP	6	→ E97_b	→ 4	itp(6 + 4)
	6	← E97_f	← 4	itp(6 + 4)
ITP	itp(itp(itp(12 + 8)<br>+ 34) + 20)	→ E96_b	→ itp(6 + 4)	itp(itp(itp(itp(12 + 8) + 34) + 20)<br>+ itp(6 + 4))
	itp(itp(itp(12 + 8)<br>+ 34) + 20)	← E96_f	← itp(6 + 4)	itp(itp(itp(itp(12 + 8) + 34) + 20)<br>+ itp(6 + 4))
ITP	itp(itp(itp(itp(12<br>+ 8) + 34) + 20)<br>+ itp(6 + 4))	→ E63_f	→ itp(itp(<br>itp(itp(68 + 42)<br>+ 41) + 40) + 39)	itp(itp(itp(itp(itp(12 + 8)<br>+ 34) + 20) + itp(6 + 4))<br>+ itp(itp(itp(itp(68 + 42) +<br>41) + 40) + 39))
	itp(itp(itp(itp(12<br>+ 8) + 34) +20)<br>+ itp(6 + 4))	← E63_b	← itp(itp(<br>itp(itp(68 + 42)<br>+ 41) + 40) + 39)	itp(itp(itp(itp(itp(12 + 8)<br>+ 34) + 20) + itp(6 + 4))<br>+ itp(itp(itp(itp(68 + 42) +<br>41) + 40) + 39))
ITP	102	→ E66 101_b	→ 101	itp(102 + 101)
	102	← E66 101_f	← 101	itp(102 + 101)
ITP	itp(102 + 101)	→ E67 95_b	→ 95	itp(itp(102 + 101) + 95)
	itp(102 + 101)	← E67 95_f	← 95	itp(itp(102 + 101) + 95)
ITP	37	→ E66 95_f	→ itp(<br>itp(102 + 101) + 95)	itp(37 + itp(itp(102 + 101) + 95))
	37	← E67 101_b	← itp(<br>itp(102 + 101) + 95)	itp(37 + itp(itp(102 + 101) + 95))
CTP	ctp(E64 + E65)	→ itp(37 + itp(itp(<br>102 + 101) + 95))	→ ctp(<br>ctp(E67 + E68)<br>+ ctp(E69 + E70))	ctp(ctp(E64 + E65) +<br>ctp(ctp(E67 + E68) +<br>ctp(E69 + E70)))
Parallel	47	→ ctp(E81_104<br>+ ctp(E82_111<br>+ E81_111))	→ 108	parallel(ctp(E81_104<br>+ ctp(E82_111 + E81_111))ctp(<br>E81_100+ ctp(E82_90 + E81_90)))
	47	→ ctp(E81_100<br>+ ctp(E82_90<br>+ E81_90))	→ 108	parallel(ctp(E81_104 + ctp(<br>E82_111 + E81_111))ctp(E81_100<br>+ ctp(E82_90 + E81_90)))
CTP	parallel(ctp(E81_<br>104 + ctp(E82_111<br>+ E81_111)) + ctp(<br>E81_100 + ctp(<br>E82_90 + E81_90<br>)))	→ 108	→ ctp(ctp(<br>E83 + E84) + E85)	ctp(parallel(ctp(E81_104<br>+ ctp(E82_111 + E81_111))<br>+ ctp(E81_100 + ctp(E82_90<br>+ E81_90))) + ctp(ctp(E83<br>+ E84) + E85))

Supplementary Table 5. Table of reductions. For the legend see Table 1.

RT	t_i/p_i	$pc/t_i, t_j$	t_j/p_j	Reduced vertex
CTP	ctp(E79+E80)	→ 47	→ ctp(parallel(ctp(E81_104 + ctp(E82_111 + E81_111)) + ctp(E81_100 + ctp(E82_90 + E81_90))) + ctp(ctp(E83 + E84) + E85))	ctp(ctp(E79 + E80) + ctp(parallel(ctp(E81_104 + ctp(E82_111 + E81_111)) + ctp(E81_100 + ctp(E82_90 + E81_90))) + ctp(ctp(E83 + E84) + E85)))
CTP	E151	→ 87	→ E152	ctp(E151 + E152)
CTP	E23	→ 88	→ E41	ctp(E23 + E41)
CTP	E30	→ 84	→ E31	ctp(E30 + E31)
CTP	ctp(E30 + E31)	→ 83	→ E32	ctp(ctp(E30 + E31) + E32)
CTP	R5_2	→ 81	→ R3	ctp(R5_2 + R3)
CTP	R5_1	→ 86	→ ctp(R5_2 + R3)	ctp(R5_1 + ctp(R5_2 + R3))
CTP	E5	→ 53	→ E4	ctp(E5 + E4)
CTP	E129_2	→ 64	→ E128	ctp(E129_2 + E128)
CTP	E122	→ 14	→ E123	ctp(E122 + E123)
CTP	E42	→ 117	→ E43	ctp(E42 + E43)
CTP	E61_119	→ 120	→ E61_80	ctp(E61_119 + E61_80)
CTP	E44	→ 119	→ ctp(E61_119 + E61_80)	ctp(E44 + ctp(E61_119 + E61_80))
CTP	ctp(E42 + E43)	→ 118	→ ctp(E44 + ctp(E61_119 + E61_80))	ctp(ctp(E42 + E43) + ctp(E44 + ctp(E61_119 + E61_80)))
CTP	E132	→ 124	→ E137	ctp(E132 + E137)
CTP	ctp(E132 + E137)	→ 125	→ E138	ctp(ctp(E132 + E137) + E138)
CTP	E98	→ 11	→ E99	ctp(E98 + E99)
CTP	E140	→ 26	→ E141	ctp(E140 + E141)
CTP	E135	→ 1	→ E40	ctp(E135 + E40)
CTP	E117	→ 91	→ E116	ctp(E117 + E116)
CTP	E112	→ 114	→ E115	ctp(E112 + E115)
CTP	ctp(E117 + E116)	→ 113	→ OUT_113	ctp(ctp(E117 + E116) + OUT_113)
CTP	E64	→ 36	→ E65	ctp(E64 + E65)
CTP	ctp(E135 + E40)	→ 25	→ E136	ctp(ctp(E135 + E40) + E136)

Supplementary Table 6. Table of reductions. For the legend see Table 1.

RT	t_i/p_i	$pc/t_i, t_j$	t_j/p_j	Reduced vertex
CTP	E69	→ 103	→ E70	ctp(E69 + E70)
CTP	E67	→ 96	→ E68	ctp(E67 + E68)
CTP	ctp(E67 + E68)	→ 97	→ ctp(E69 + E70)	ctp(ctp(E67 + E68) + ctp(E69 + E70))
CTP	E83	→ 7	→ E84	ctp(E83 + E84)
CTP	E82_90	→ 90	→ E81_90	ctp(E82_90 + E81_90)
CTP	E82_111	→ 111	→ E81_111	ctp(E82_111 + E81_111)
CTP	E81_100	→ 100	→ ctp(E82_90 + E81_90)	ctp(E81_100 + ctp(E82_90 + E81_90))
CTP	E81_104	→ 104	→ ctp(E82_111 + E81_111)	ctp(E81_104 + ctp(E82_111 + E81_111))
CTP	E79	→ 112	→ E80	ctp(E79 + E80)
CTP	E36_f	→ 75	→ E36_b	ctp(E36_f + E36_b)
CTP	E95	→ 55	→ OUT_55_sinapate-esther-synthesis	ctp(E95 + OUT_55_sinapate-esther-synthesis)
CTP	E86_31	→ 31	→ E87_48	ctp(E86_31 + E87_48)
CTP	E88_19	→ 30a	→ OUT_30a	ctp(E88_19 + OUT_30a)
CTP	ctp(E83 + E84)	→ 17	→ E85	ctp(ctp(E83 + E84) + E85)
CTP	E86_133	→ 133	→ E87_134	ctp(E86_133 + E87_134)
CTP	E88_48	→ 30b	→ OUT_30b	ctp(E88_48 + OUT_30b)
CTP	E74	→ 99	→ OUT_99	ctp(E74 + OUT_99)
ITP	130<br>130	→ E143_f<br>← E143_b	→ 78<br>← 78	itp(130 + 78)<br>itp(130 + 78)
ITP	82<br>82	→ E20_f<br>← E20_b	→ 77<br>← 77	itp(82 + 77)<br>itp(82 + 77)
ITP	76<br>76	→ E24_f<br>← E24_b	→ 50<br>← 50	itp(76 + 50)<br>itp(76 + 50)
ITP	69<br>69	→ E34_b<br>← E34_f	→ 56<br>← 56	itp(69 + 56)<br>itp(69 + 56)
ITP	49<br>49	→ spo_b<br>← spo_f	→ itp(69 + 56)<br>← itp(69 + 56)	itp(49 + itp(69 + 56))<br>itp(49 + itp(69 + 56))

Supplementary Table 7. Table of reductions. For the legend see Table 1.

RT	t_i/p_i	$pc/t_i, t_j$	t_j/p_j	Reduced vertex
ITP	itp(49 + itp(69 + 56))	→ E8_f	→ 44	itp(itp(49 + itp(69 + 56)) + 44)
	itp(49 + itp(69 + 56))	← E8_b	← 44	itp(itp(49 + itp(69 + 56)) + 44)
ITP	itp(itp(49 + itp(69 + 56)) + 44)	→ E9_f	→ itp(76 + 50)	itp(itp(itp(49 + itp(69+56)) + 44) + itp(76 + 50))
	itp(itp(49 + itp(69 + 56)) + 44)	← E9_b	← itp(76 + 50)	itp(itp(itp(49 + itp(69 + 56)) + 44) + itp(76 + 50))
ITP	itp(itp(itp(49 + itp(69 + 56)) + 44) + itp(76 + 50))	→ R6_f	→ 89	itp(itp(itp(itp(49 + itp(69 + 56)) + 44) + itp(76+50))+89)
	itp(itp(itp(49 + itp(69 + 56)) + 44) + itp(76 + 50))	← R6_b	← 89	itp(itp(itp(itp(49 + itp(69 + 56)) + 44) + itp(76 + 50)) + 89)
ITP	itp(itp(itp(itp(49 + itp(69 + 56)) + 44) + itp(76 + 50)) + 89)	→ E16	→ 43	itp(itp(itp(itp(itp(49 + itp(69 + 56)) + 44) + itp(76 + 50)) + 89) + 43)
	itp(itp(itp(itp(49 + itp(69 + 56)) + 44) + itp(76 + 50)) + 89)	← E15	← 43	itp(itp(itp(itp(itp(49 + itp(69 + 56)) + 44) + itp(76 + 50)) + 89) + 43)
ITP	68	→ E13_f	→ 42	itp(68 + 42)
	68	← E13_b	← 42	itp(68 + 42)
ITP	itp(68 + 42)	→ E91_f	→ 41	itp(itp(68 + 42) + 41)
	itp(68 + 42)	← E91_b	← 41	itp(itp(68 + 42) + 41)
ITP	itp(itp(68 + 42) + 41)	→ E90_f	→ 40	itp(itp(itp(68 + 42) + 41) + 40)
	itp(itp(68 + 42) + 41)	← E90_b	← 40	itp(itp(itp(68 + 42) + 41) + 40)
ITP	45	→ E148_f	→ 52	itp(45 + 52)
	45	← E148_b	← 52	itp(45 + 52)
ITP	itp(itp(itp(itp(itp(49 + itp(69 + 56)) + 44) + itp(76 + 50)) + 89) + 43)	→ E6_f	→ 54	itp(itp(itp(itp(itp(itp(49 + itp(69 + 56)) + 44) + itp(76 + 50)) + 89) + 43) + 54)
	itp(itp(itp(itp(itp(49 + itp(69 + 56)) + 44) + itp(76 + 50)) + 89) + 43)	← E6_b	← 54	itp(itp(itp(itp(itp(itp(49 + itp(69 + 56)) + 44) + itp(76 + 50)) + 89) + 43) + 54)
ITP	itp(45 + 52)	→ E3_b	→ 46	itp(itp(45 + 52) + 46)
	itp(45 + 52)	← E3_f	← 46	itp(itp(45 + 52) + 46)
ITP	itp(itp(itp(68 + 42) + 41) + 40)	→ E89_f	→ 39	itp(itp(itp(itp(68 + 42) + 41) + 40) + 39)
	itp(itp(itp(68 + 42) + 41) + 40)	← E89_b	← 39	itp(itp(itp(itp(68 + 42) + 41) + 40) + 39)
ITP	129	→ E139_u_ttod	→ 115	itp(129 + 115)
	129	← E144_u	← 115	itp(129 + 115)

Supplementary Table 8. Table of reductions. For the legend see Table 1.

RT	t_i/p_i	$pc/t_i, t_j$	t_j/p_j	Reduced vertex
ITP	itp(129 + 115)	→ E139_u_dtom	→ 80	itp(itp(129 + 115) + 80)
	itp(129 + 115)	← E62_u	← 80	itp(itp(129 + 115) + 80)
ITP	126	→ E35_u	→ itp(itp(129 + 115) + 80)	itp(126 + itp(itp(129 + 115) + 80))
	126	← E120_	← itp(itp(129 + 115) + 80)	itp(126 + itp(itp(129 + 115) + 80))
ITP	128	→ E139_c_dtom	→ 123	itp(128 + 123)
	128	← E62_c	← 123	itp(128 + 123)
ITP	122	→ E139_c_ttod	→ itp(128 + 123)	itp(122 + itp(128 + 123))
	122	← E144_c	← itp(128 + 123)	itp(122 + itp(128 + 123))
ITP	itp(122 + itp(128 + 123))	→ E120_c	→ 127	itp(itp(122 + itp(128 + 123)) + 127)
	itp(122 + itp(128 + 123))	← E35_c	← 127	itp(itp(122 + itp(128 + 123)) + 127)
ITP	12	→ E94_b	→ 8	itp(12 + 8)
	12	← E94_f	← 8	itp(12 + 8)
ITP	itp(12 + 8)	→ E93	→ 34	itp(itp(12 + 8) + 34)
	itp(12 + 8)	← E92	← 34	itp(itp(12 + 8) + 34)
ITP	itp(itp(12 + 8) + 34)	→ E111	→ 20	itp(itp(itp(12 + 8) + 34) + 20)
	itp(itp(12 + 8) + 34)	← E110	← 20	itp(itp(itp(12 + 8) + 34) + 20)
ITP	6	→ E97_b	→ 4	itp(6 + 4)
	6	← E97_f	← 4	itp(6 + 4)
ITP	itp(itp(itp(12 + 8) + 34) + 20)	→ E96_b	→ itp(6 + 4)	itp(itp(itp(itp(12 + 8) + 34) + 20) + itp(6 + 4))
	itp(itp(itp(12 + 8) + 34) + 20)	← E96_f	← itp(6 + 4)	itp(itp(itp(itp(12 + 8) + 34) + 20) + itp(6 + 4))
ITP	itp(itp(itp(itp(12 + 8) + 34) + 20) + itp(6 + 4))	→ E63_f	→ itp(itp(itp(itp(68 + 42) + 41) + 40) + 39)	itp(itp(itp(itp(itp(12 + 8) + 34) + 20) + itp(6 + 4)) + itp(itp(itp(itp(68 + 42) + 41) + 40) + 39))
	itp(itp(itp(itp(12 + 8) + 34) + 20) + itp(6 + 4))	← E63_b	← itp(itp(itp(itp(68 + 42) + 41) + 40) + 39)	itp(itp(itp(itp(itp(12 + 8) + 34) + 20)+ itp(6 + 4)) + itp(itp(itp(itp(68 + 42) + 41) + 40) + 39))
ITP	102	→ E66_101_b	→ 101	itp(102 + 101)
	102	← E66_101_f	← 101	itp(102 + 101)

Supplementary Table 9. Table of reductions. For the legend see Table 1.

RT	t_i/p_i	$pc/t_i, t_j$	t_j/p_j	Reduced vertex
ITP	itp(102 + 101)<br>itp(102 + 101)	→ E67_95_b<br>← E67_95_f	→ 95<br>← 95	itp(itp(102 + 101) + 95)<br>itp(itp(102 + 101) + 95)
ITP	37<br>37	→ E66_95_f<br>← E67_101_b	→ itp(itp(102 + 101) + 95)<br>← itp(itp(102 + 101) + 95)	itp(37 + itp(itp(102 + 101) + 95))<br>itp(37 + itp(itp(102 + 101) + 95))
CTP	ctp(E64 + E65)	→ itp(37 + itp(itp(102 + 101) + 95))	→ ctp(ctp(E67 + E68) + ctp(E69 + E70))	ctp(ctp(E64 + E65) + ctp(ctp(E67 + E68) + ctp(E69 + E70)))
Parallel	47<br>47	→ ctp(E81_104 + ctp(E82_111 + E81_111))<br>→ ctp(E81_100 + ctp(E82_90 + E81_90))	→ 108<br>→ 108	parallel(ctp(E81_104 + ctp(E82_111 + E81_111))ctp(E81_100 + ctp(E82_90 + E81_90)))<br>parallel(ctp(E81_104 + ctp(E82_111 + E81_111))ctp(E81_100 + ctp(E82_90 + E81_90)))
CTP	parallel(ctp(E81_104 + ctp(E82_111 + E81_111)) + ctp(E81_100 + ctp(E82_90 + E81_90)))	→ 108	→ ctp(ctp(E83 + E84) + E85)	ctp(parallel(ctp(E81_104 + ctp(E82_111 + E81_111)) + ctp(E81_100 + ctp(E82_90 + E81_90))) + ctp(ctp(E83 + E84) + E85))
CTP	ctp(E79 + E80)	→ 47	→ ctp(parallel(ctp(E81_104 + ctp(E82_111 + E81_111)) + ctp(E81_100 + ctp(E82_90 + E81_90))) + ctp(ctp(E83 + E84) + E85))	ctp(ctp(E79 + E80) ctp(parallel(ctp(E81_104 + ctp(E82_111 + E81_111)) + ctp(E81_100 + ctp(E82_90 + E81_90))) + ctp(ctp(E83 + E84) + E85)))
